# Supplementary material for: Biomimetic chiral hydrogen-bonded organic-inorganic frameworks
Source: Nat Commun. 2024 Jan 2;15:139. doi: 10.1038/s41467-023-43700-6 (PMC10762213; doi:10.1038/s41467-023-43700-6)
Supplement: Supplementary file 1 — Supplementary Information [file 41467_2023_43700_MOESM1_ESM.pdf]

Supplementary Information for

Biomimetic chiral hydrogen-bonded organic-inorganic framework

Jun Guo[1★], Yulong Duan[1], Yunling Jia[2], Zelong Zhao[1], Xiaoqing Gao[3], Pai Liu[2], Fangfang Li[1], Hongli Chen[2], Yutong Ye[2], Yujiao Liu[1], Meiting Zhao[4★], Zhiyong Tang[5★] and Yi Liu[1★]

[1]State Key Laboratory of Separation Membranes and Membrane Processes, School of Chemistry, Tiangong University, Tianjin 300387, P. R. China.

[2]School of Materials Science and Engineering, Tiangong University, Tianjin 300387, P. R. China.

[3]Wenzhou Key Laboratory of Biomaterials and Engineering, Wenzhou Institute, University of Chinese Academy of Sciences, Wenzhou 325000, P. R. China.

[4]Tianjin Key Laboratory of Molecular Optoelectronic Sciences, Department of Chemistry, Institute of Molecular Aggregation Science, Tianjin University, Tianjin 300072, P. R. China.

[5]CAS Key Laboratory of Nanosystem and Hierarchical Fabrication, CAS Center for Excellence in Nanoscience, National Center for Nanoscience and Technology, Beijing 100190, P. R. China.

★Corresponding author: junguo@tiangong.edu.cn, mtzhao@tju.edu.cn, yiliuchem@whu.edu.cn, zytang@nanoctr.cn

Table of Contents

Supplementary Figures Page 3

Supplementary Tables Page 52

Supplementary References Page 72

Supplementary Figures

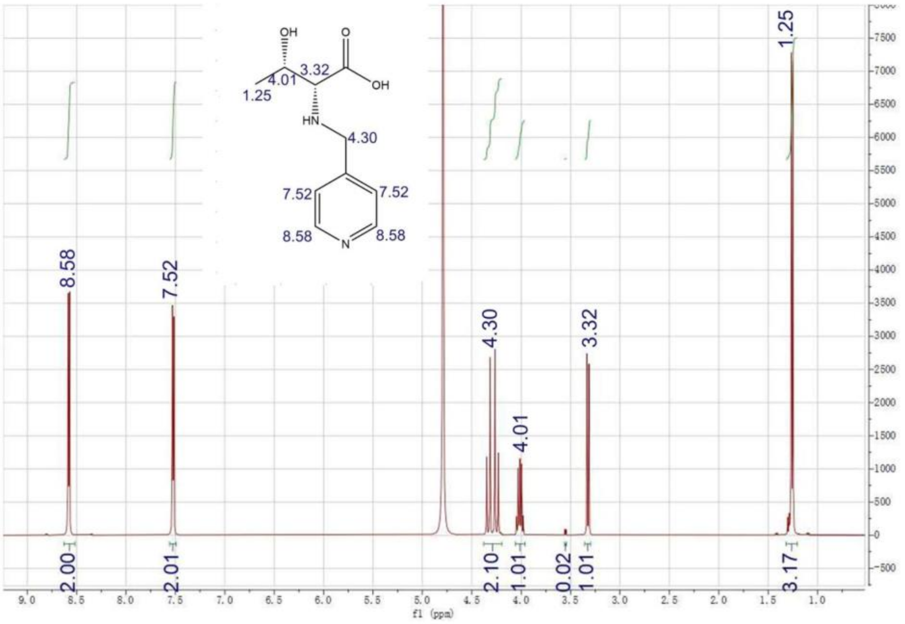

Supplementary Figure 1. ^{1}H-NMR spectrum of *d*-thr. (The peak centered at 4.79 ppm is the residual solvent peak of D_2O).

Peak assignments: ^{1}H-NMR (D_2O, ppm): $-CH_3$ (1.25, d, 3H), -HN-CH (3.32, d, 1H), -CH (4.01, m, 1H), $-CH_2$ (4.30 dd, 2H), py-H (7.52, d, 2H), py-H (8.58, d, 2H). The ^{1}H-NMR spectrum demonstrates the successful synthesis of *d*-thr in high purity.

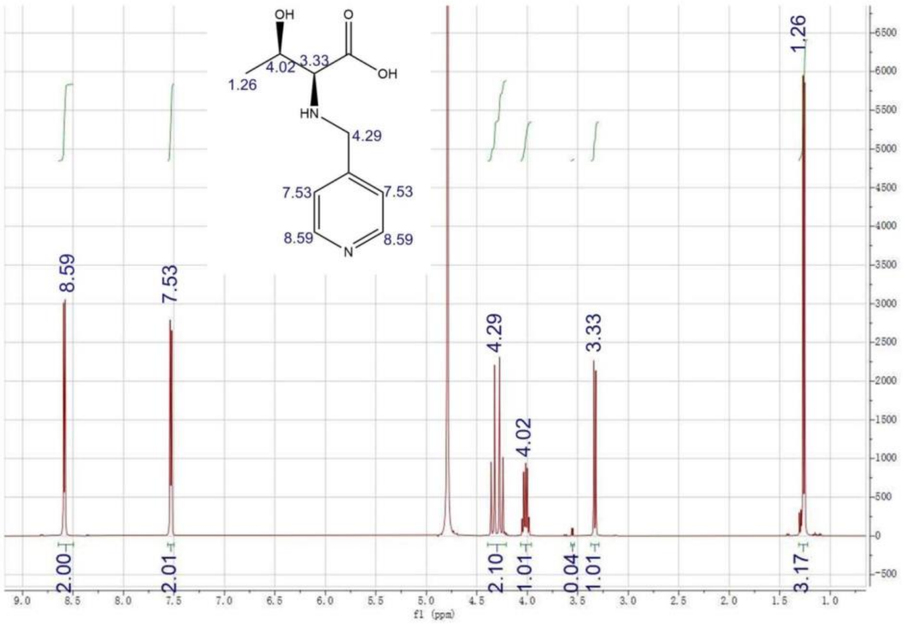

Supplementary Figure 2. ^{1}H-NMR spectrum of *l*-thr. (The peak centered at 4.79 ppm is the residual solvent peak of D_2O).

Peak assignments: ^{1}H-NMR (D_2O, ppm): -CH_3 (1.26, d, 3H), -HN-CH (3.33, d, 1H), -CH (4.02, m, 1H), -CH_2 (4.29 dd, 2H), py-H (7.53, d, 2H), py-H (8.59, d, 2H). The ^{1}H-NMR spectrum demonstrates the successful synthesis of *l*-thr in high purity.

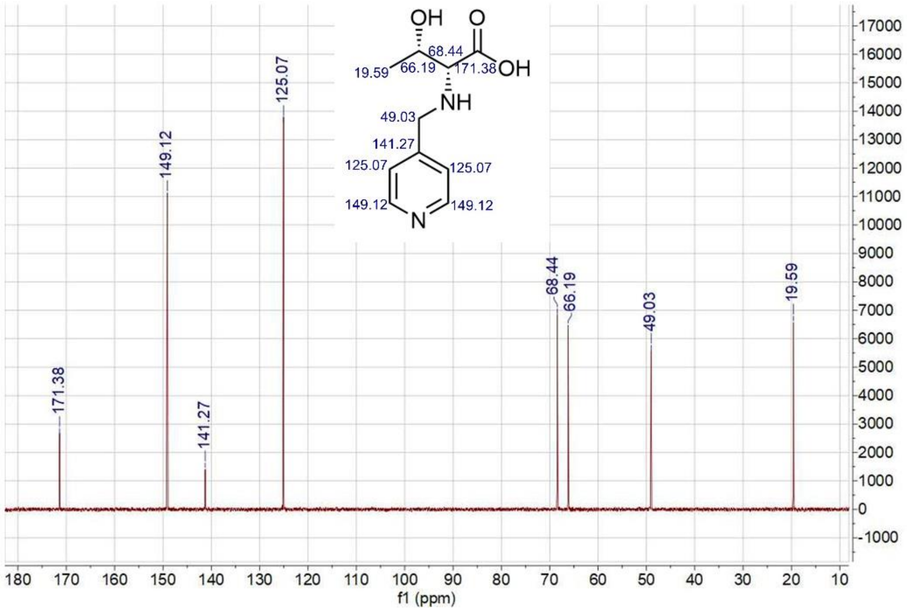

Supplementary Figure 3. ^{13}C-NMR spectrum of *d*-thr.

Peak assignments: ^{13}C-NMR (solid sample, ppm): -CH_3 (19.59), -CH_2 (49.03), -CH-OH (66.19), -CH-HN (68.44), py-C (125.07), py-C (141.27), py-C (149.12), -COOH (171.38). The ^{13}C-NMR spectrum confirms the successful synthesis of *d*-thr in high purity.

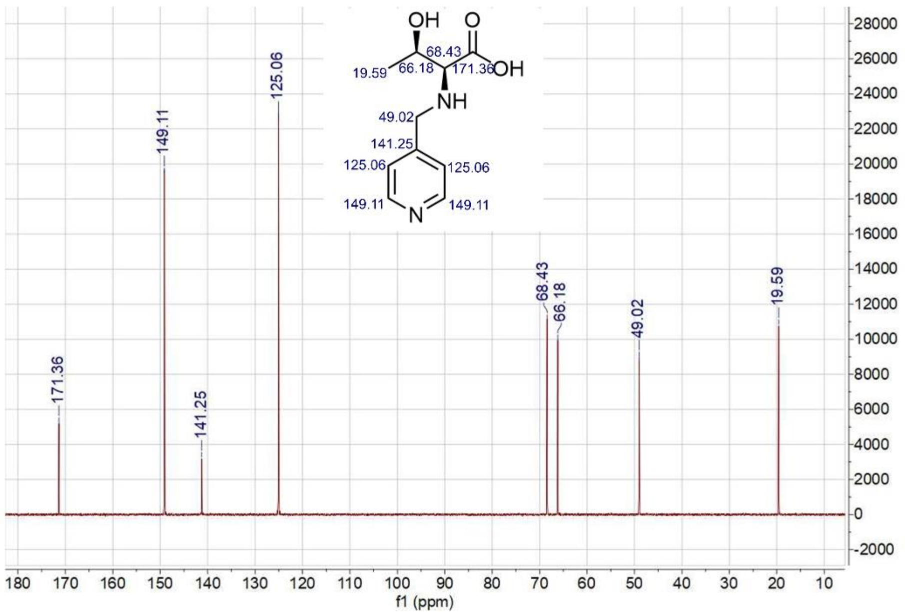

Supplementary Figure 4. ^{13}C-NMR spectrum of *l*-thr.

Peak assignments: ^{13}C-NMR (solid sample, ppm): -CH_3 (19.59), -CH_2 (49.03), -CH-OH (66.18), -CH-HN (68.43), py-C (125.06), py-C (141.25, py-C (149.11), -COOH (171.36). The ^{13}C-NMR spectrum confirms the successful synthesis of *l*-thr in high purity.

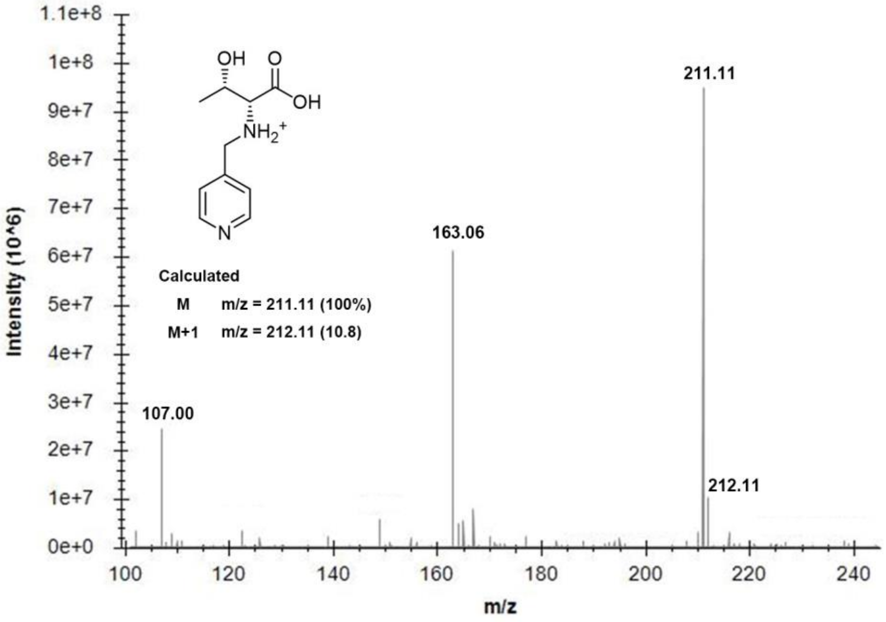

Supplementary Figure 5. MS spectrum of *d*-thr.

As shown above, the peak of m/z = 211.11 is the molecular ion peak of protonated *d*-thr and the peak of m/z = 212.11 is the corresponding isotopic signal. The consistency between the observed data with the calculated ones confirms the successful synthesis of *d*-thr ligand.

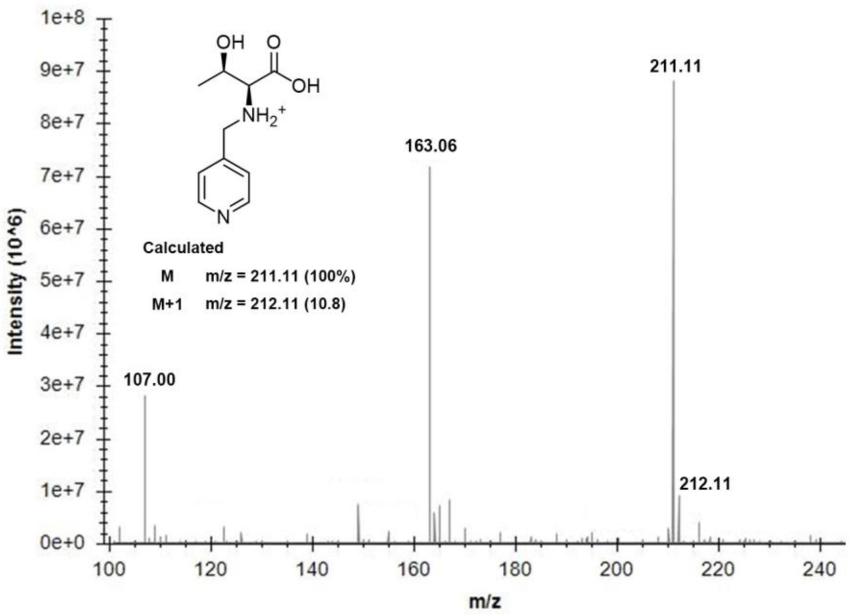

Supplementary Figure 6. MS spectrum of *l*-thr.

Similarly, the peak of m/z = 211.11 is the molecular ion peak of protonated *l*-thr and the peak of m/z = 212.11 is the corresponding isotopic signal. The consistency between the observed data with the calculated ones confirms the successful synthesis of *l*-thr ligand.

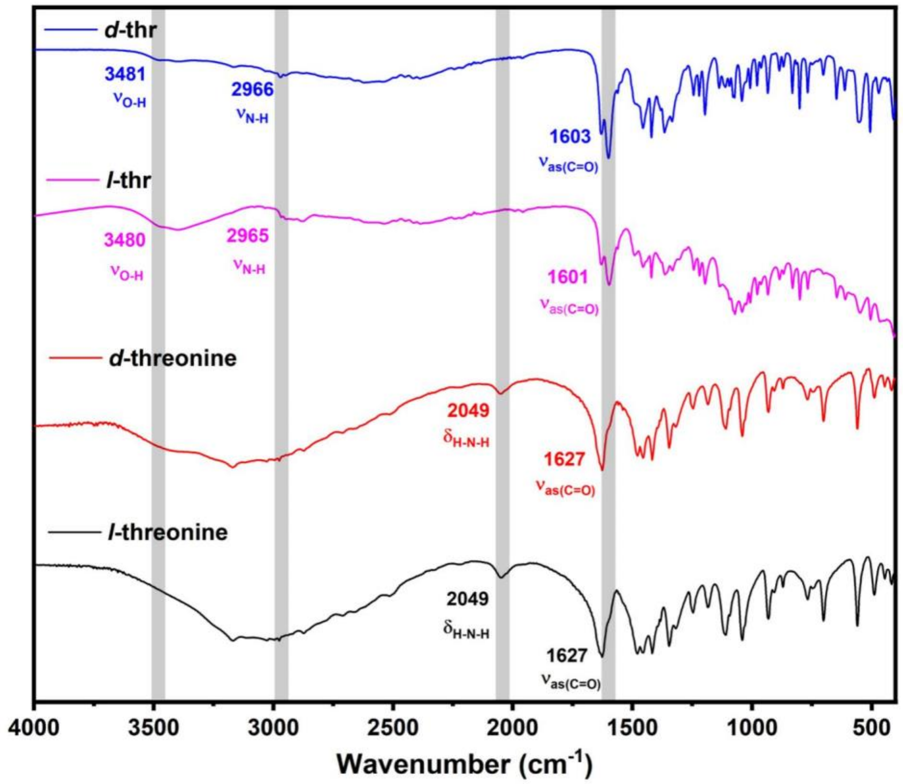

Supplementary Figure 7. FT-IR spectra of *d*-thr (blue curve), *l*-thr (purple curve), *d*-threonine (red curve) and *l*-threonine (black curve).

Compared with *d*(*l*)-threonine precursor, the disappearance of H-N-H bending absorption peak centered at 2049 cm^{-1} proves the successful formation of *d*(*l*)-thr ligands via Schiff-base condensation that is sequentially reduced to the secondary amine (ν_{N-H}, 2966 cm^{-1}). Moreover, the asymmetric stretching vibration of carboxyl group of *d*(*l*)-thr ligands moves to 1603 cm^{-1}, which is also different from 1627 cm^{-1} of raw *d*(*l*)-threonine.

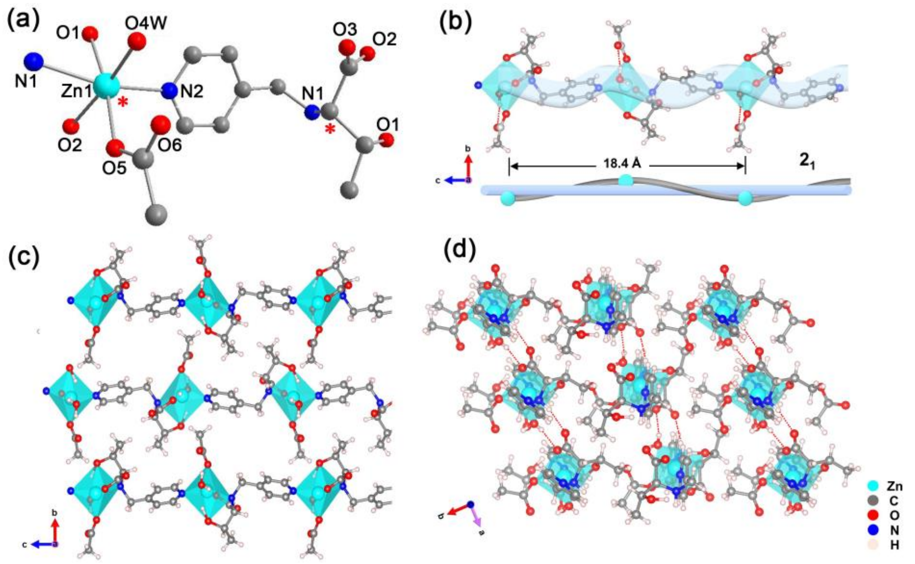

Supplementary Figure 8. **(a)** Coordination mode of Zn(II) ion in *l*-Zn-HOIF. **(b)** 1D helical chain structure of *l*-Zn-HOIF projected along the *a*-axis. **(c)** 2D structure of *l*-Zn-HOIF projected within the *b*-*c* plane. **(d)** 3D framework of *l*-Zn-HOIF viewed down the *c*-axis.

The crystal structure was depictured by noncommercial visualization software.[1-2] As the image shown in Supplementary Fig. 8a, *l*-Zn-HOIF presents the same assemble fashion but opposite chiral configuration as that of *d*-Zn-HOIF (Fig. 2a). In detail, Zn(II) center exhibits a 6-coordinated asymmetric mode with one pyridine nitrogen from *l*-thr, one amine nitrogen, one hydroxyl oxygen and one carboxylate oxygen from another *l*-thr, and additionally one acetate oxygen and one water. In comparison to the left-handed helix (Fig. 2b), the resultant 1D spiral chain with *l*-thr adopts a right-handed 2_1 screw with a pitch of 18.4 Å along the *c*-axis (Supplementary Fig. 8b). Furthermore, these 1D helices form an ordered 2D sheet structure within the *b*-*c* plane (Supplementary Fig. 8c). Thanks to additional hydrogen bonding between adjacent sheets (O_WH···O-C, 1.867 Å and N-H···O-C, 1.980 Å, Supplementary Fig. 8d), the 3D framework is finally built by the AA stacking of 2D sheets along the *a*-axis.

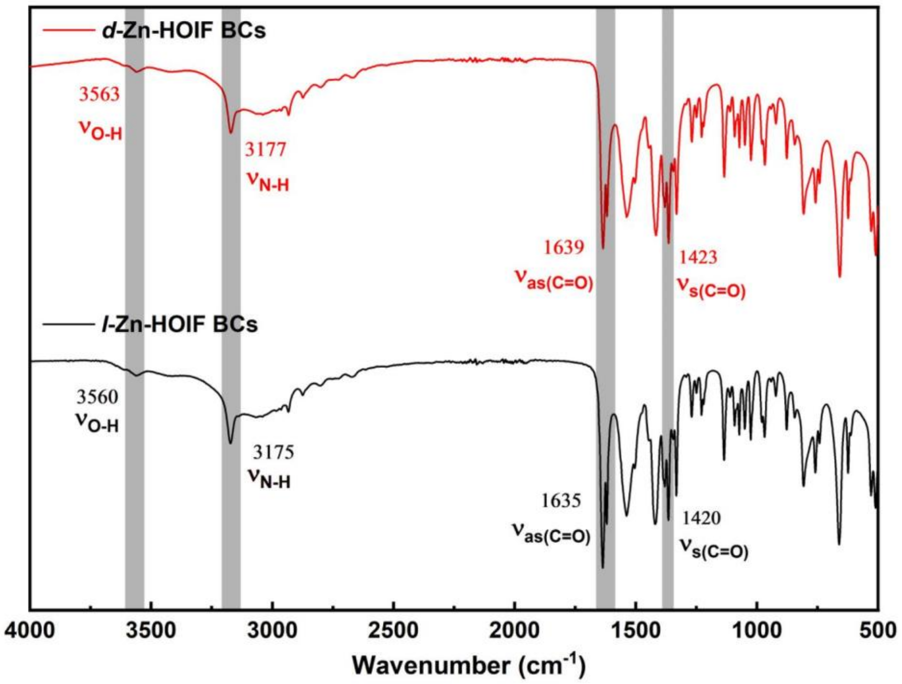

Supplementary Figure 9. FT-IR spectra of *d*-Zn-HOIF BCs (red curve) and *l*-Zn-HOIF BCs (black curve).

As-synthesized *d*-Zn-HOIF BCs and *l*-Zn-HOIF BCs present nearly the same FT-IR absorption spectra. Taking the *d*-Zn-HOIF BCs as a representative, the peak centered at 3563 cm^{-1} is assigned to the stretching vibration of -OH and the peak centered at 3177 cm^{-1} is attributed to the stretching vibration of -NH. While the peaks centered at 1639 cm^{-1} and 1423 cm^{-1} are ascribed to the asymmetric and symmetric stretching vibration of carboxyl group, respectively.

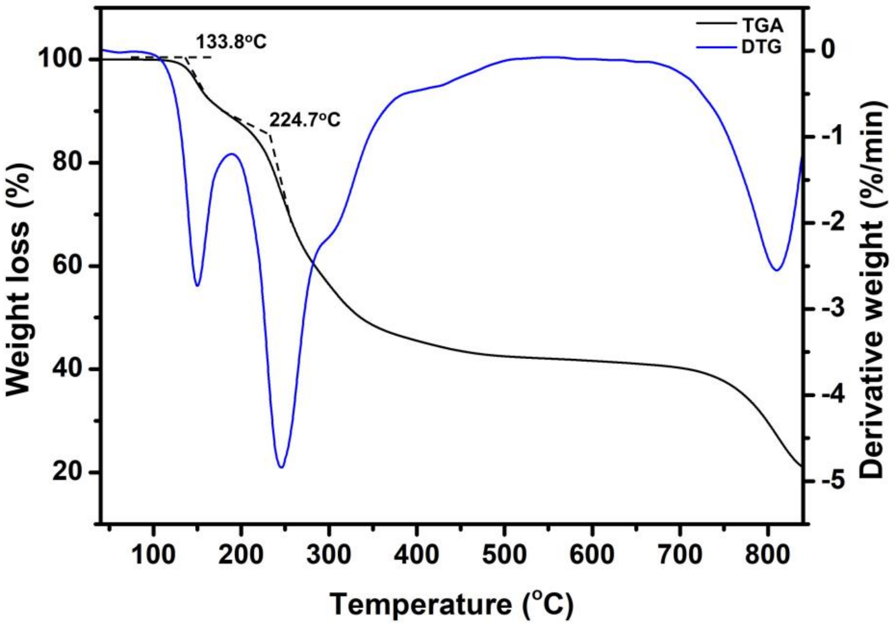

Supplementary Figure 10. TG curve (black) and derivative thermogravimetric analysis (DTG) curve (blue) of *d*-Zn-HOIF BCs.

On basis of the above result, *d*-Zn-HOIF BCs begin to lose the solvent molecules and coordinated water molecules at 133.8 ºC. As the temperature rises to 224.7 ºC, the framework begins to decompose by losing organic ligands.

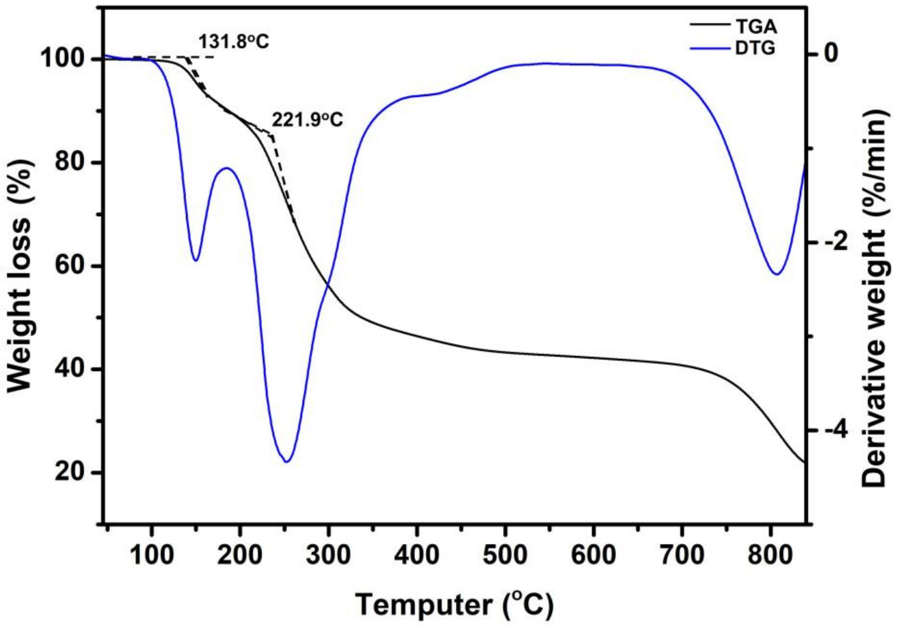

Supplementary Figure 11. TG curve (black) and DTG curve (blue) of *l*-Zn-HOIF BCs.

On basis of the above result, *l*-Zn-HOIF BCs begin to lose the solvent molecules and coordinated water molecules at 131.8 ºC. As the temperature rises to 221.9 ºC, the framework begins to decompose by losing organic ligands.

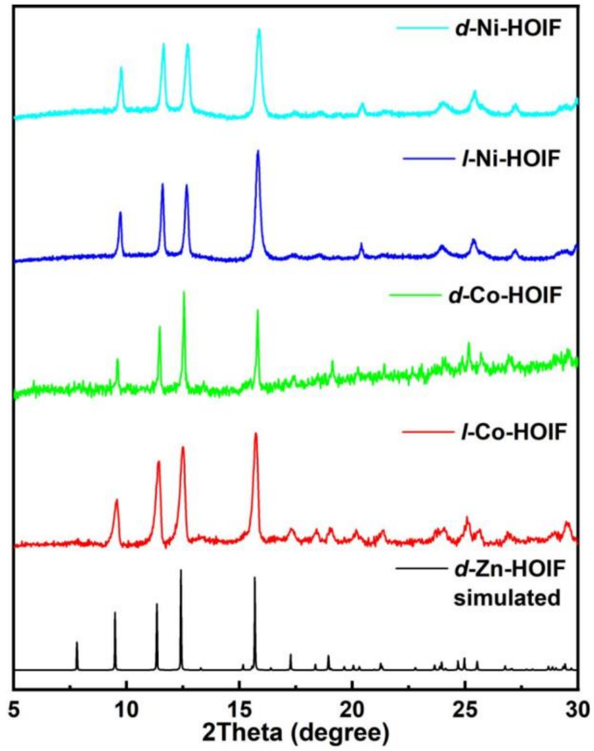

Supplementary Figure 12. The PXRD plots of *d*(*l*)-Co-HOIFs and *d*(*l*)-Ni-HOIFs.

Clearly, *d*(*l*)-Co-HOIFs and *d*(*l*)-Ni-HOIFs show nearly the same PXRD pattern as that of Zn-HOIFs, confirming their isoreticular framework structures.

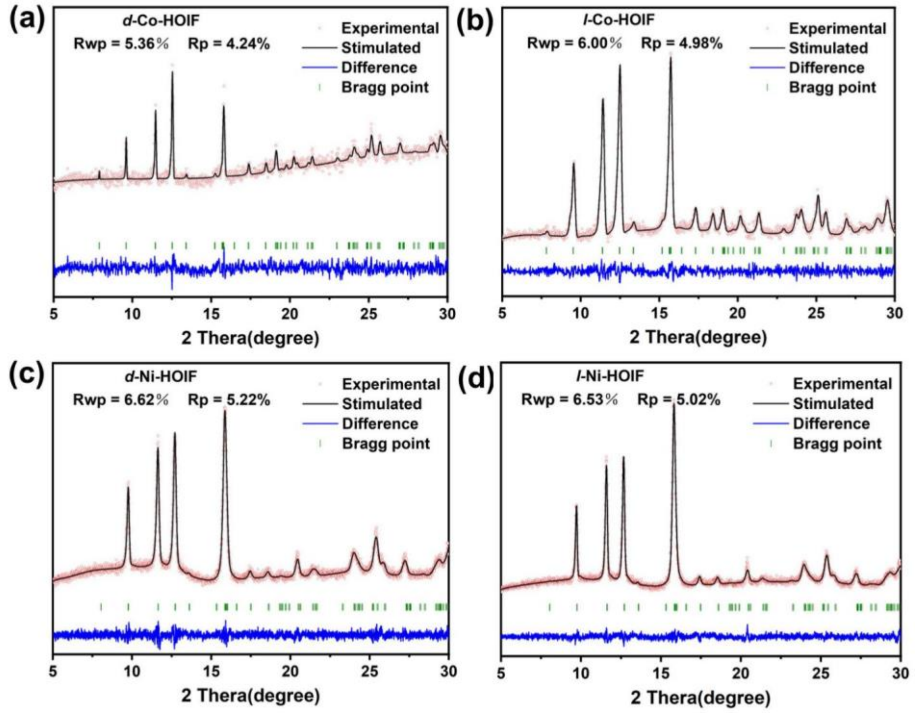

Supplementary Figure 13. PXRD refinement patterns of **(a)** *d*-Co-HOIFs, **(b)** *l*-Co-HOIFs, **(c)** *d*-Ni-HOIFs and **(d)** *l*-Ni-HOIFs.

Considering their isoreticular structures, PXRD patterns of *d*(*l*)-Co-HOIFs and *d*(*l*)-Ni-HOIFs have been refined by the Pawley refinement method in order to obtain exact cell parameters.

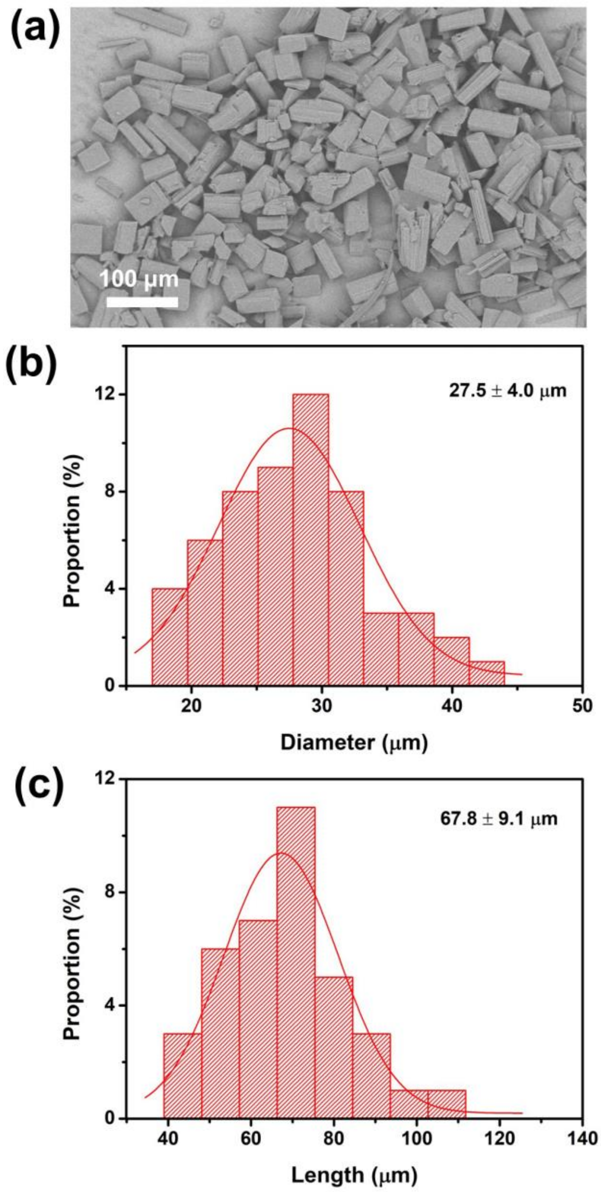

Supplementary Figure 14. **(a)** SEM image of *d*-Zn-HOIF BCs. **(b)** Diameter distribution of *d*-Zn-HOIF BCs. **(c)** Length distribution of *d*-Zn-HOIF BCs.

According to the statistical counting, the *d*-Zn-HOIF BCs present an average diameter of 27.5 μm and an average length of 67.8 μm.

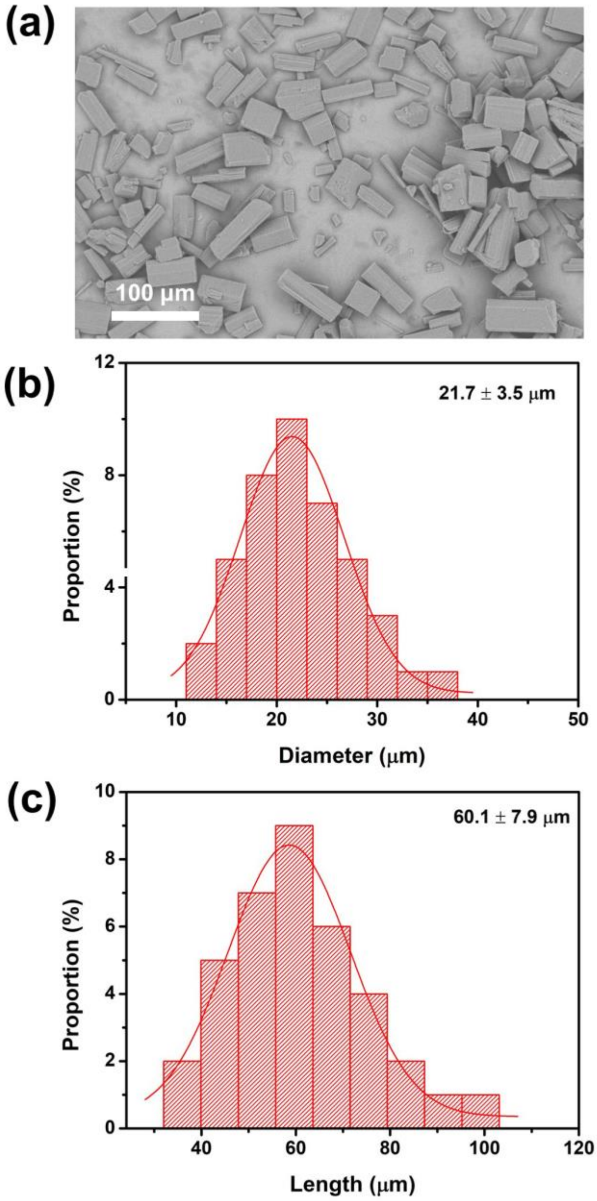

Supplementary Figure 15. **(a)** SEM image of *l*-Zn-HOIF BCs. **(b)** Diameter distribution of *l*-Zn-HOIF BCs. **(c)** Length distribution of *l*-Zn-HOIF BCs.

According to the statistical counting, the *l*-Zn-HOIF BCs present an average diameter of 21.7 μm and an average length of 60.1 μm.

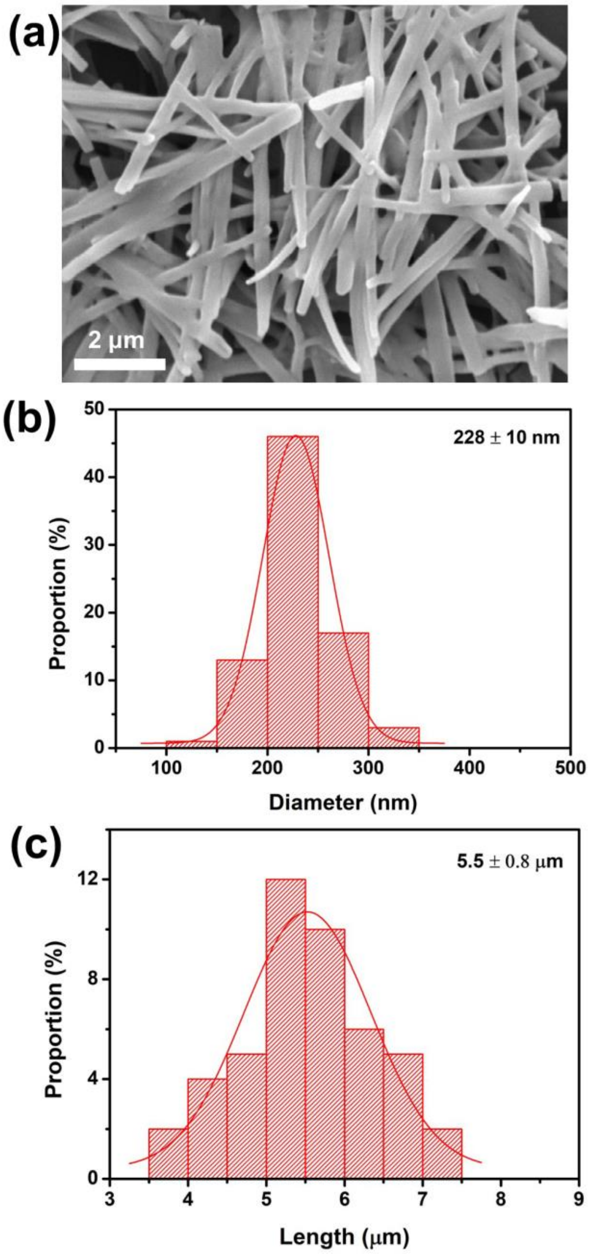

Supplementary Figure 16. **(a)** SEM image of *d*-Zn-HOIF NFs. **(b)** Diameter distribution of *d*-Zn-HOIF NFs. **(c)** Length distribution of *d*-Zn-HOIF NFs.

According to the statistical counting, the *d*-Zn-HOIF NFs present an average diameter of 228 nm and an average length of 5.5 μm.

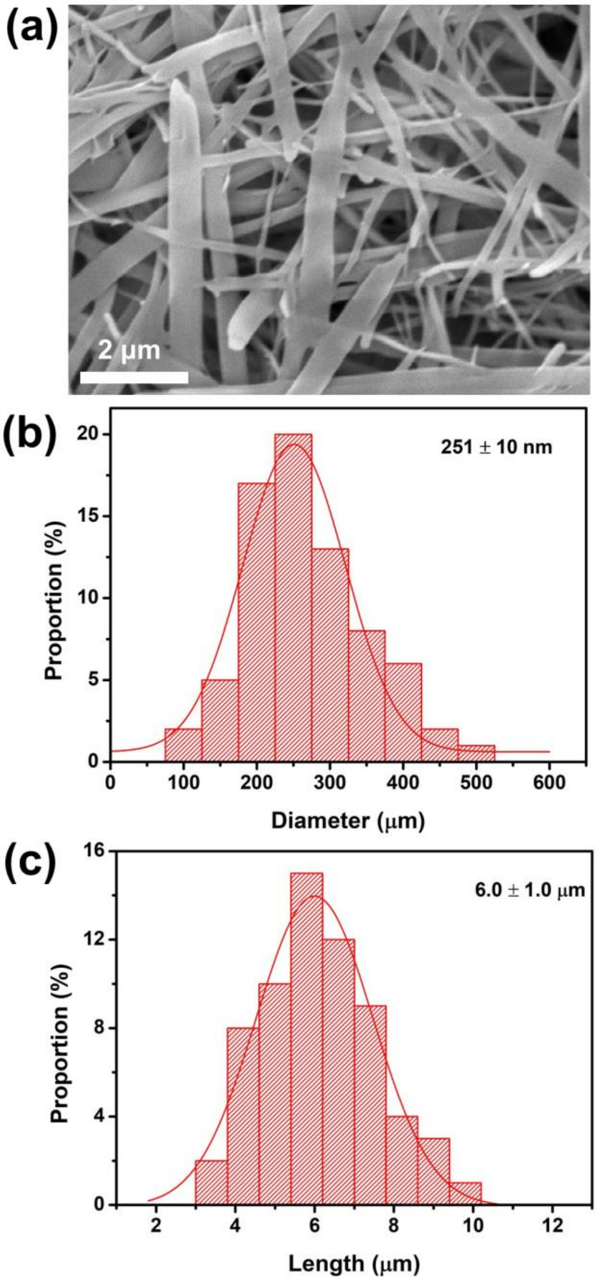

Supplementary Figure 17. **(a)** SEM image of *l*-Zn-HOIF NFs. **(b)** Diameter distribution of *l*-Zn-HOIF NFs. **(c)** Length distribution of *l*-Zn-HOIF NFs.

According to the statistical counting, the *l*-Zn-HOIF NFs present an average diameter of 186 nm and an average length of 6.0 μm.

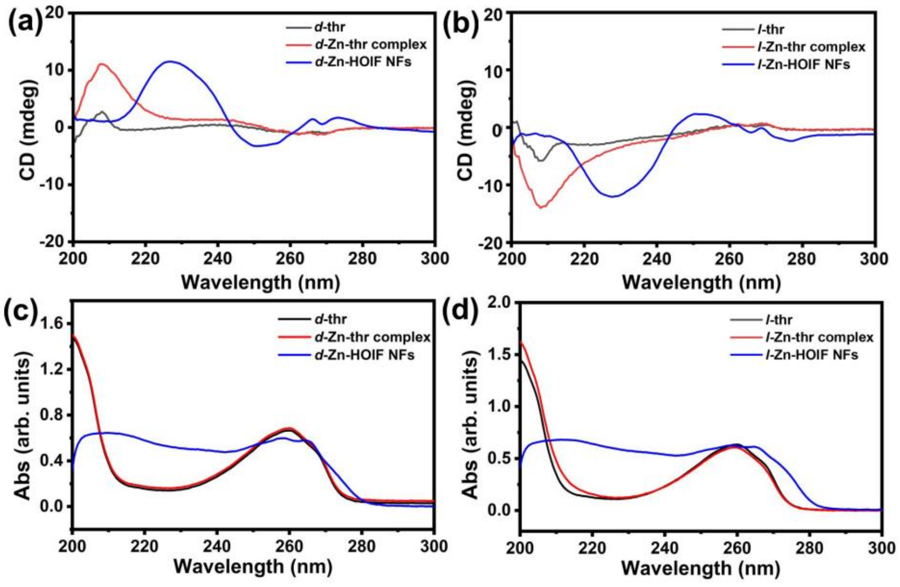

Supplementary Figure 18. **(a)** CD spectra comparison of observed *d*-thr, *d*-Zn-thr complex and *d*-Zn-HOIFs, **(b)** CD spectra comparison of observed *l*-thr, *l*-Zn-thr complex and *l*-Zn-HOIFs, **(c)** and **(d)** corresponding absorbance spectra.

As shown in Supplementary Fig. 18, both *d*(*l*)-Zn-HOIFs have showed absolutely distinct chiroptical responses in the absorption range of 200-300 nm compared with *d*(*l*)-thr and *d*(*l*)-Zn-thr complex. Specifically, the CD responses within 240-280 nm observed from *d*(*l*)-Zn-HOIFs (blue curves) merely are assigned to the pyridyl chromophore that is involved in the unique hierarchically helical structure of HOIFs. In sharp contrast, the neglectable CD signals within this region reflect the insufficient chiroptical induction of only chiral carbon centers in both *d*(*l*)-thr (black curves) and *d*(*l*)-Zn-thr complex (red curves).

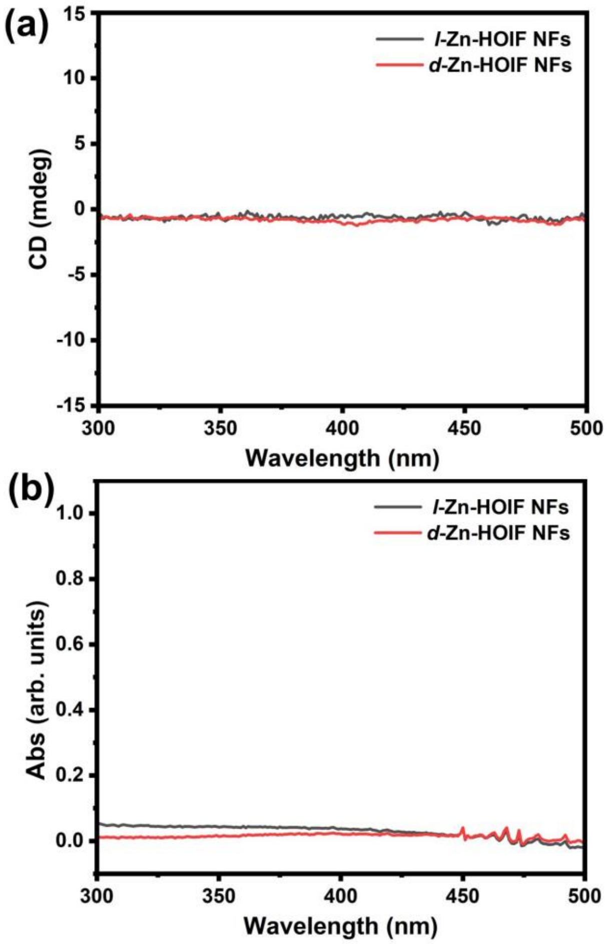

Supplementary Figure 19. **(a)** CD and **(b)** absorption spectrum of *d*(*l*)-Zn-HOIFs in the wavelength region from 300 to 500 nm.

There is no CD response in the longer wavelengths for *d*(*l*)-Zn-HOIFs as their absent absorbance in the corresponding region.

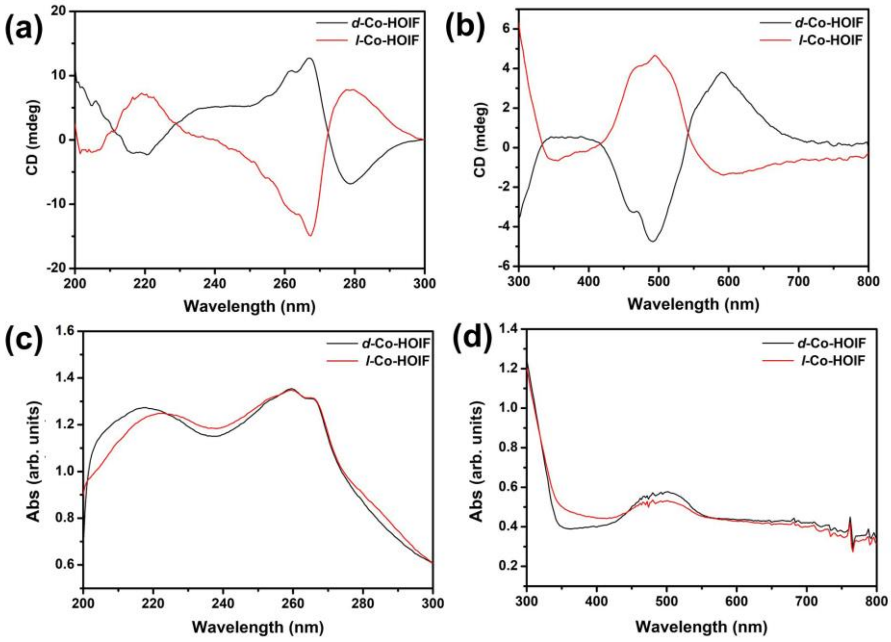

Supplementary Figure 20. **(a)** Solid CD spectra of *d*- and *l*-Co-HOIF in 200 to 300 nm. **(b)** Solid CD spectra of *d*- and *l*-Co-HOIF in 300 to 800 nm. **(c)** Corresponding UV absorption spectra of *d*- and *l*-Co-HOIF in 200 to 300 nm. **(d)** Corresponding UV absorption spectra of *d*- and *l*-Co-HOIF in 300 to 800 nm.

As seen from Supplementary Fig. 20, *d*-Co-HOIF and *l*-Co-HOIF show obvious CD responses in the UV region as well. Besides, additional ligand-to-metal charge transfer (LMCT) process occurred for *d*(*l*)-Co-HOIFs in 300 to 800 nm thanks to the not fully occupied $3d^7$ orbitals of Co^{2+} center. Obviously, the strong and mirror-symmetric CD signals in 300 to 800 nm further demonstrate the chiroptical activities of *d*(*l*)-Co-HOIFs at visible light regions.

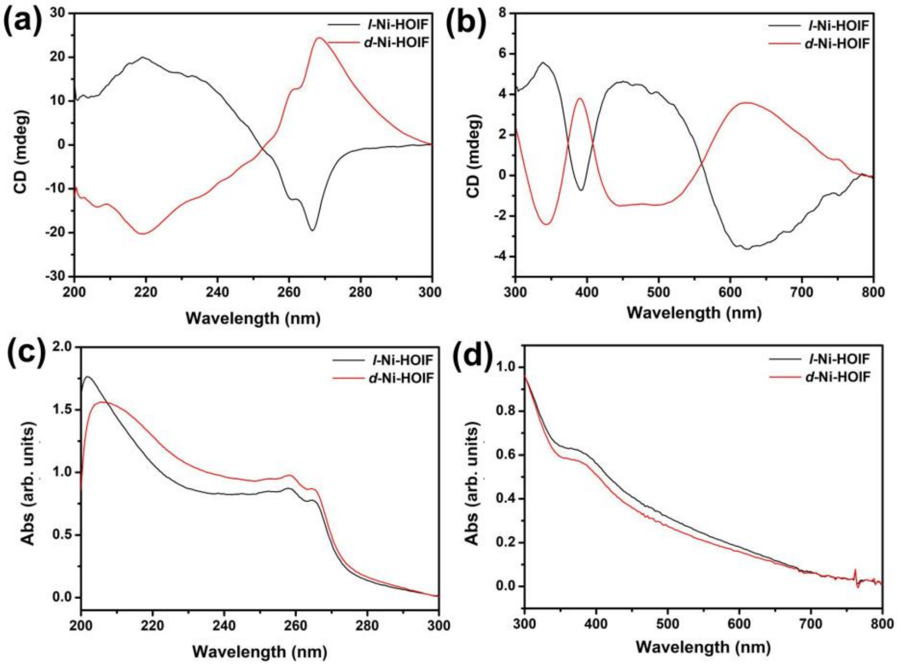

Supplementary Figure 21. **(a)** Solid CD spectra of *d*-Ni-HOIF and *l*-Ni-HOIF in 200 to 300 nm. **(b)** Solid CD spectra of *d*-Ni-HOIF and *l*-Ni-HOIF in 300 to 800 nm. **(c)** Corresponding UV absorption spectra of *d*-Ni-HOIF and *l*-Ni-HOIF in 200 to 300 nm. **(d)** Corresponding UV absorption spectra of *d*-Ni-HOIF and *l*-Ni-HOIF in 300 to 800 nm.

As seen from Supplementary Fig. 21, *d*-Ni-HOIF and *l*-Ni-HOIF show obvious CD responses in the UV region as well. Besides, similar LMCT process occurred for *d*(*l*)-Ni-HOIFs in 300 to 800 nm thanks to the not fully occupied $3d^8$ orbitals of Ni^{2+} center. Hence, the strong and mirror-symmetric CD signals in 300 to 800 nm further demonstrate the chiroptical activities of *d*(*l*)-Ni-HOIFs at visible light regions.

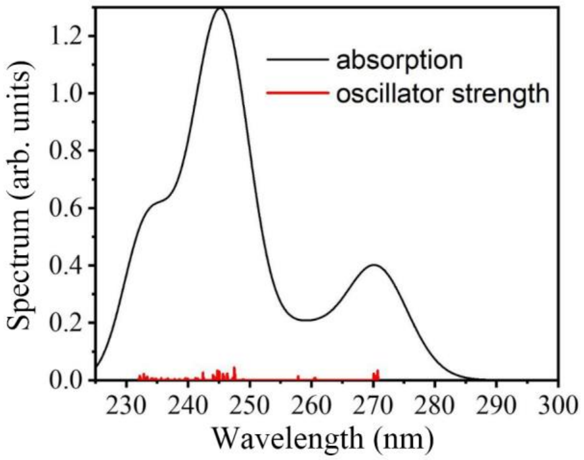

Supplementary Figure 22. The calculated absorption spectra of *d*-Zn-HOIF.

As result shown in Supplementary Fig. 22, the calculated absorption spectrum of *d*-Zn-HOIF presents the lowest absorption gap around 270 nm which is in line with the experimentally obtained absorbance (Fig. 3c) and declares the good reliability of our calculation in return.

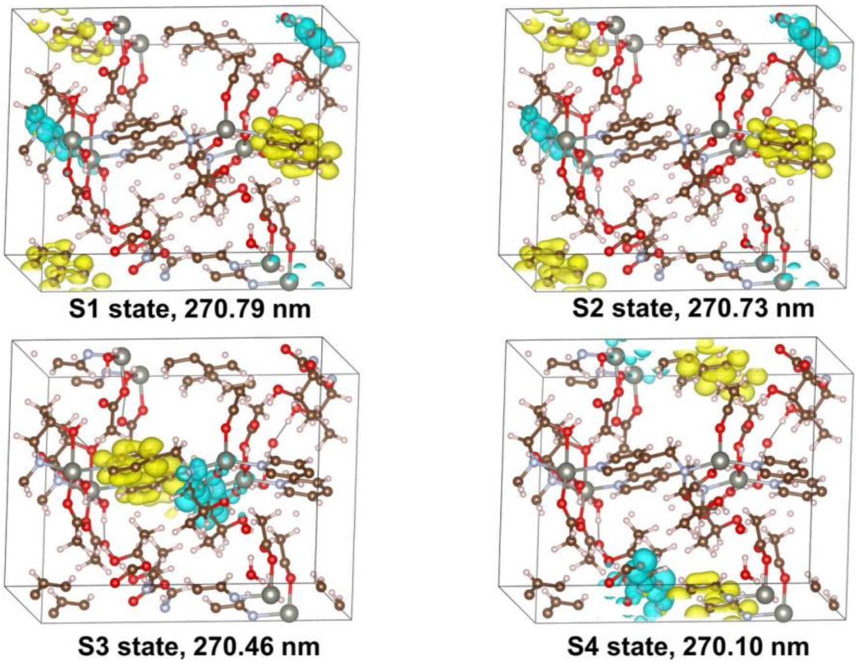

Supplementary Figure 23. The lowest four excited states of *d*-Zn-HOIF. The color shadow indicates the electron transition from the cyan to yellow region.

The electron transition density maps for corresponding excited states are drawn in Supplementary Fig. 23 for detailed analysis. Obviously, the involved four excited states coherently present an intramolecular charge transfer (ICT) transition between pyridyl with neighboring chemosphere (as shadows indicated electron transitions from the cyan to yellow region). In principle, ICT renders a large red-shift for the emission band of a molecule caused by the concomitant dissipation of excitation energy that can be as high as 1.0 eV.[3-4]

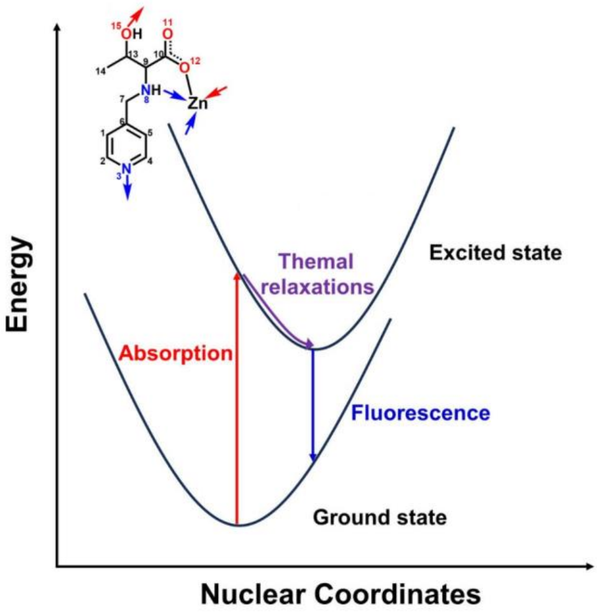

Supplementary Figure 24. Schematic illustration of rotation and distortion relaxations of the excited state based on Franck-Condon principle.

Thermal relaxations of the excited state also greatly contribute to the Stokes shift on basis of the Franck-Condon principle. Especially for a fluorophore substituted with flexible groups via free single bonds (e.g., the C7-N8 single bond in our case, Supplementary Fig. 24), large rotation and distortion relaxations around such single bond also result in large Stokes shift.[5-6]

Overall, the large Stokes shift ($\Delta\lambda$ = ca. 130 nm) observed in our case is reasoned to the ICT-induced dissipation and unique thermal relaxations of excited states. The reasonability of our reported data has been further confirmed by the large Stokes shift reported in literature about similar pyridyl-derived fluorophore.[7-8]

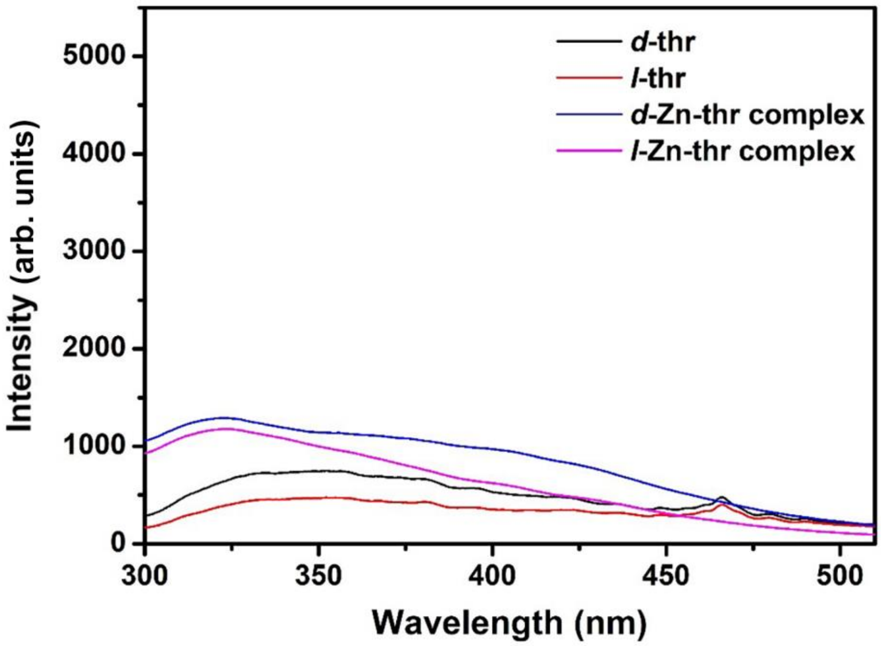

Supplementary Figure 25. Fluorescence spectra of *d*(*l*)-thr and *d*(*l*)-Zn-thr complex.

As shown in Supplementary Fig. 25, *d*(*l*)-thr ligands and *d*(*l*)-Zn-thr complex show negligible emission.

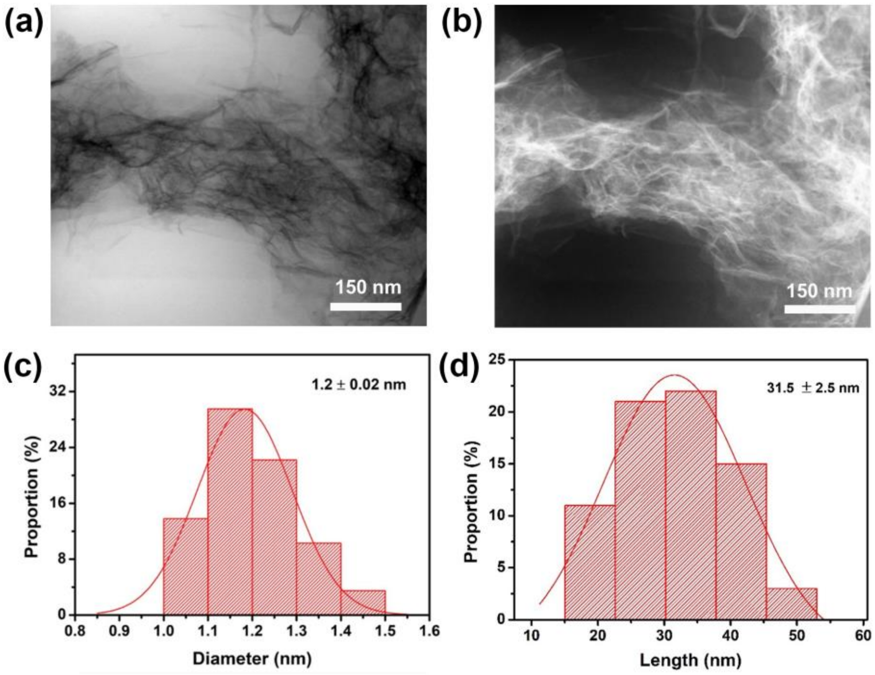

Supplementary Figure 26. **(a)** HR-TEM image of *d*-Zn-HOIF DSs in H_2O. **(b)** HAAD-STEM image of *d*-Zn-HOIF DSs in H_2O. **(c)** Diameter distribution of *d*-Zn-HOIF DSs. **(d)** Length distribution of *d*-Zn-HOIF DSs.

According to the statistical counting, the *d*-Zn-HOIF DSs in water present an average diameter of 1.2 nm and an average length of 31.5 nm.

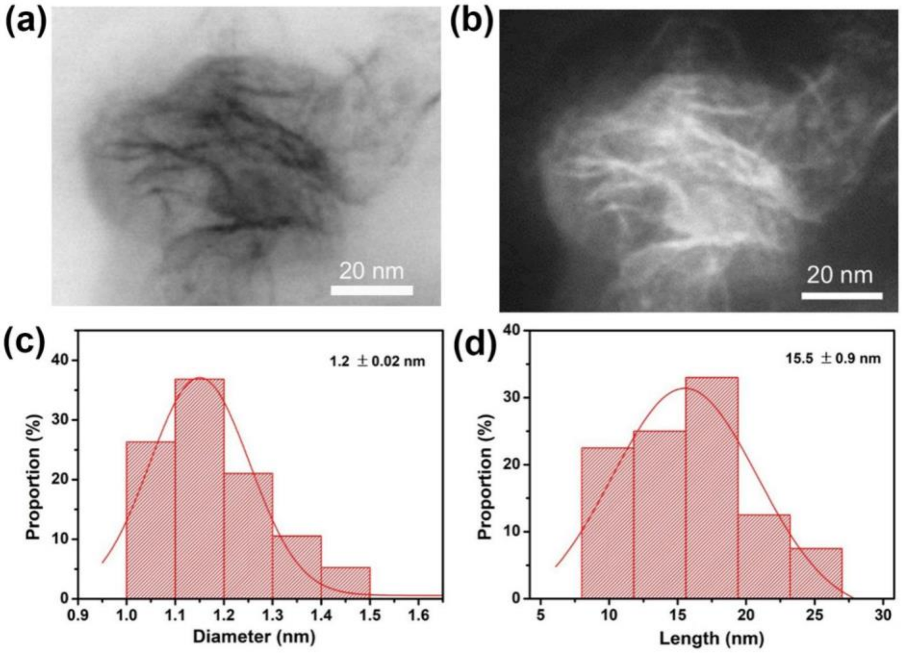

Supplementary Figure 27. **(a)** HR-TEM image of *d*-Co-HOIF DSs in H_2O. **(b)** HAAD-STEM image of *d*-Co-HOIF DSs in H_2O. **(c)** Diameter distribution of *d*-Co-HOIF DSs. **(d)** Length distribution of *d*-Co-HOIF DSs.

According to the statistical counting, the *d*-Co-HOIF DSs in water present an average diameter of 1.2 nm and an average length of 15.5 nm.

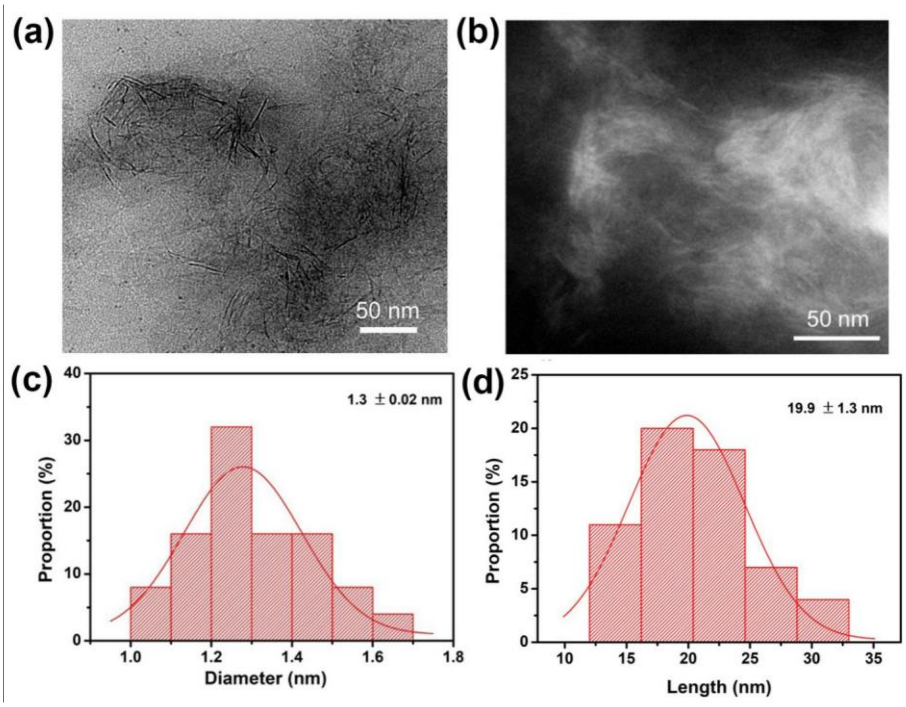

Supplementary Figure 28. **(a)** HR-TEM image of *d*-Ni-HOIF DSs in H_2O. **(b)** HAAD-STEM image of *d*-Ni-HOIF DSs in H_2O. **(c)** Diameter distribution of *d*-Ni-HOIF DSs. **(d)** Length distribution of *d*-Ni-HOIF DSs.

According to the statistical counting, the *d*-Ni-HOIF DSs in water present an average diameter of 1.3 nm and an average length of 19.9 nm.

The measured widths of both *d*-Co-HOIF DSs and *d*-Ni-HOIF DSs are in good agreement with the width of single-strand chain in crystallography. Consequently, isoreticular Co-HOIF DSs and Ni-HOIF DSs are composed of single helical stands similar to Zn-HOIF DSs.

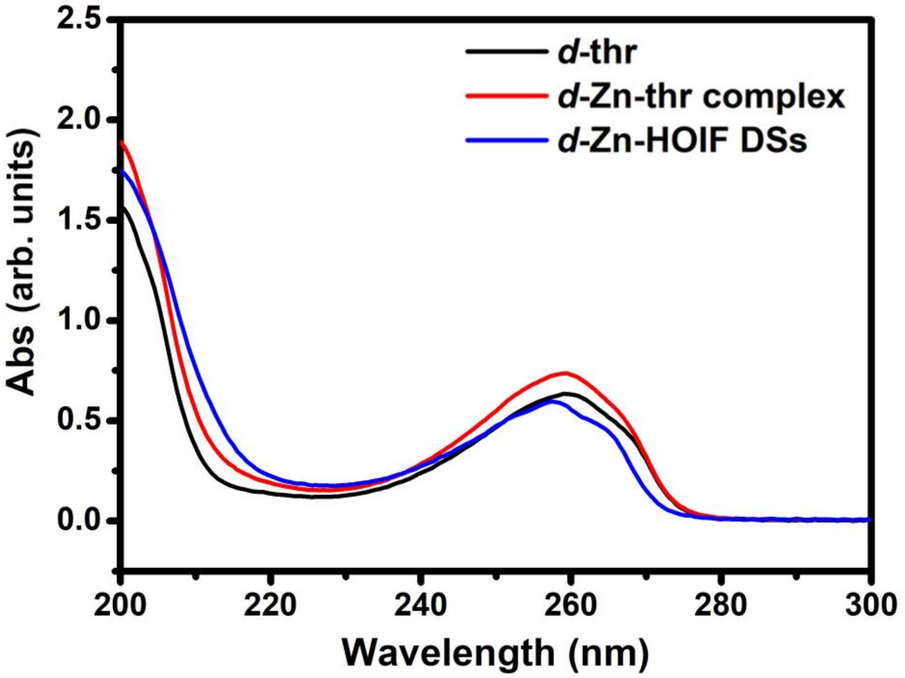

Supplementary Figure 29. UV absorption of *d*-thr, *d*-Zn-thr complex and *d*-Zn-HOIF DSs under the same concentration (5 mM) of *d*-thr motif.

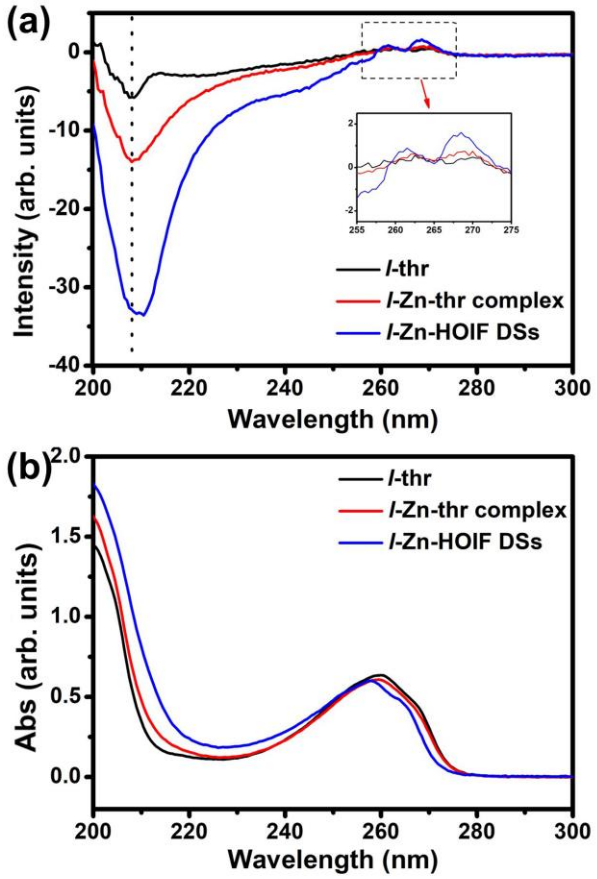

Supplementary Figure 30. **(a)** CD spectra and **(b)** UV absorption of raw *l*-thr, molecular *l*-Zn-thr complex and *l*-Zn-HOIF DSs in H_2O under the same concentration (5 mM) of *d*-thr motif. The inset magnifies the optical activity in the absorption window of aromatic pyridyl units.

Compared with CD responses of *l*-thr and *l*-Zn-thr complex, the much stronger CD signals together with the characteristic CD signals (255-275 nm) of pyridyl units verify the well-maintained 1D helical structures of *l*-Zn-HOIF DSs in H_2O.

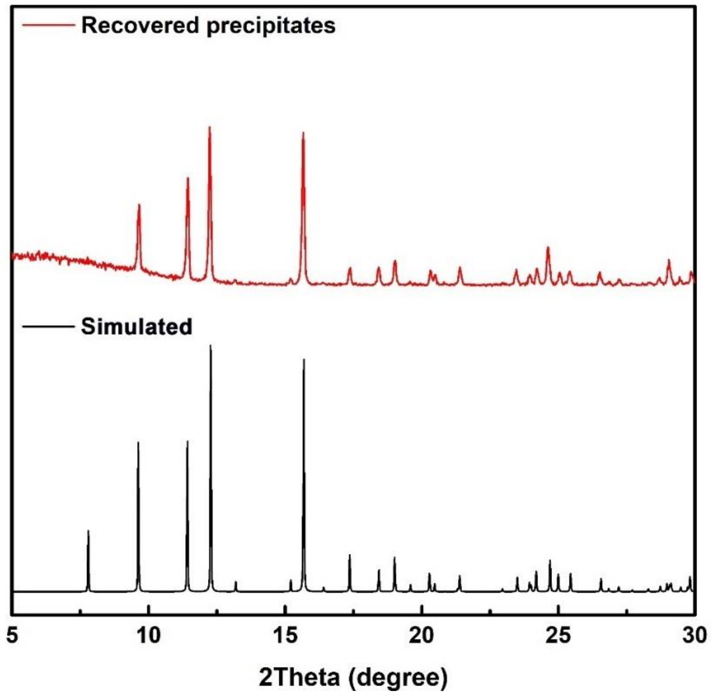

Supplementary Figure 31. PXRD curve of recovered *d*-Zn-HOIF NFs precipitates.

Distinct from the normally obtained amorphous aggregates, the sharp PXRD pattern of recovered *d*-Zn-HOIF NFs precipitates proves the ordered reassembly via reversible hydrogen bonding.

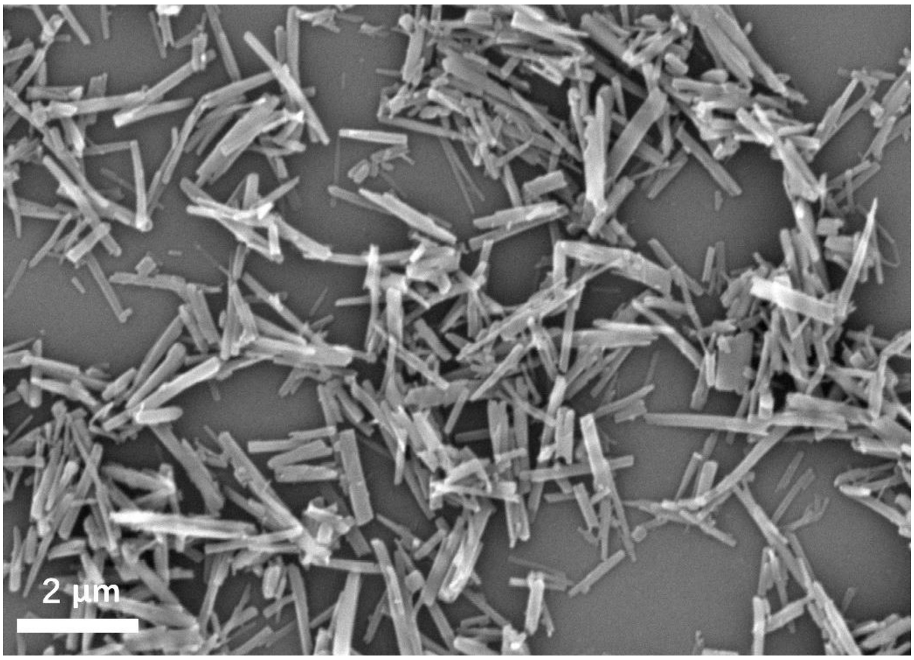

Supplementary Figure 32. SEM image of recovered *d*-Zn-HOIF precipitates.

As shown in Supplementary Fig. 32, the reassembled *d*-Zn-HOIF precipitates show the well-dispersed 1D rod morphology clearly.

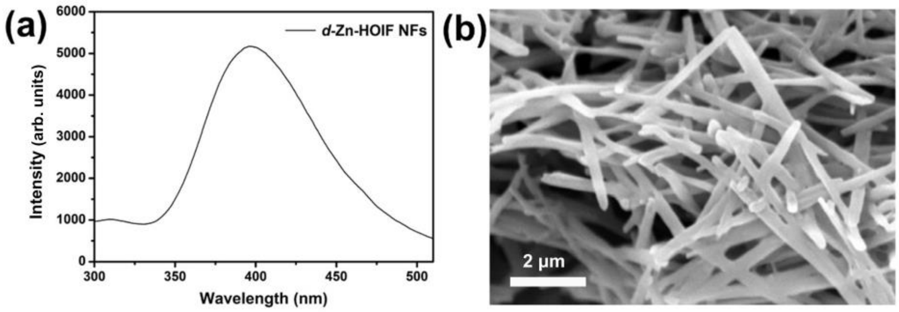

Supplementary Figure 33. **(a)** Fluorescence spectrum and **(b)** SEM image of *d*-Zn-HOIF NFs dispersed in EtOH.

Unlike disassembly in strong protic water or MeOH, *d*-Zn-HOIF NFs dispersed in EtOH well maintain their fluorescence and fibrous morphology.

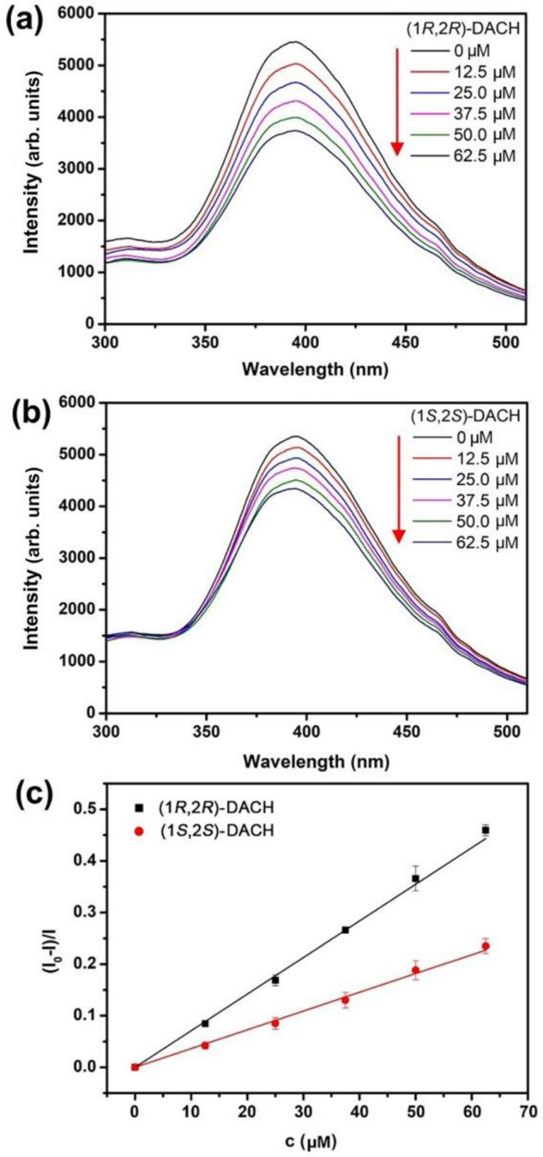

Supplementary Figure 34. Fluorescence quenching spectra of *d*-Zn-HOIF NFs by adding **(a)** (1*R*,2*R*)- and **(b)** (1*S*,2*S*)-DACH. **(c)** The corresponding SV plots. The concentration of (1*R*,2*R*)- and (1*S*,2*S*)-DACH is changed from 0 to 62.5 μM. Error bars represent standard deviations of three independent replicates.

According to the plots shown in Supplementary Fig. 34c, the K_{sv} of *d*-Zn-HOIF NFs for (1*R*,2*R*)- and (1*S*,2*S*)-DACH is fitted to be 7.3×10^3 and 3.6×10^3 M^{-1}, respectively. As a result, the QR is calculated to be 2.0.

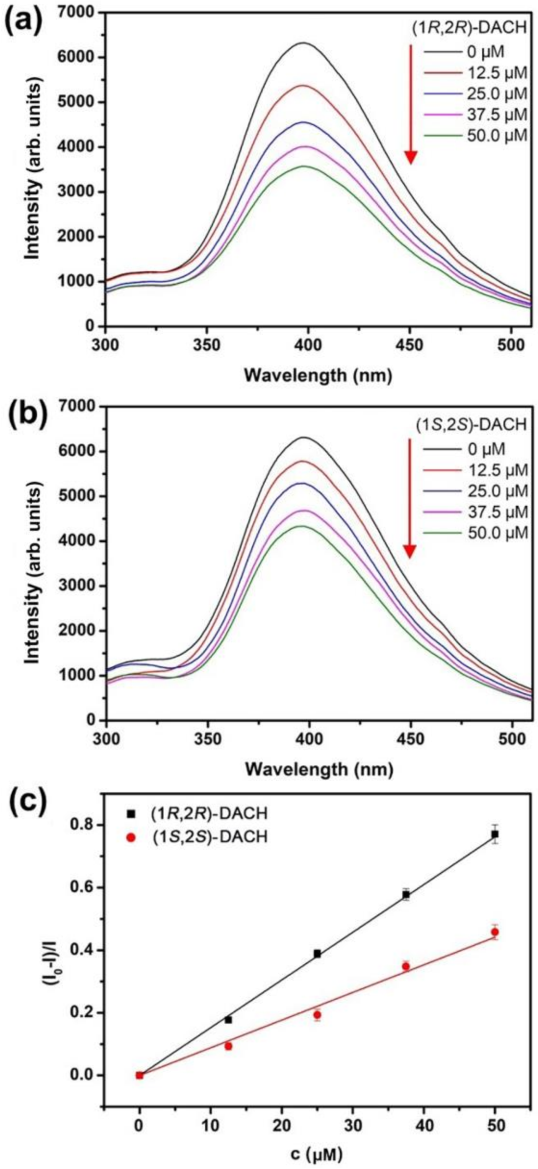

Supplementary Figure 35. Fluorescence quenching spectra of *d*-Zn-HOIF BCs by adding **(a)** (1*R*,2*R*)- and **(b)** (1*S*,2*S*)-DACH. **(c)** The corresponding SV plots. The concentration of (1*R*,2*R*)- and (1*S*,2*S*)-DACH is changed from 0 to 50 μM. Error bars represent standard deviations of three independent replicates.

According to the plots shown in Supplementary Fig. 35c, the K_{sv} of *d*-Zn-HOIF BCs for (1*R*,2*R*)- and (1*S*,2*S*)-DACH is 1.5×10^4 and 8.8×10^3 M^{-1}, respectively. As a result, the QR is calculated to be 1.7, which is lower than 2.0 of *d*-Zn-HOIF NFs.

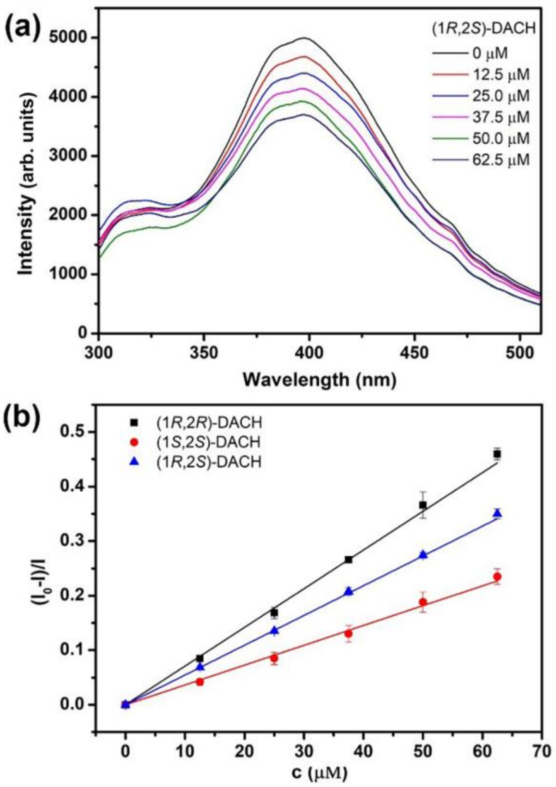

Supplementary Figure 36. **(a)** Fluorescence quenching spectra and **(b)** corresponding SV plot of *d*-Zn-HOIF NFs by adding (1*R*,2*S*)-DACH. The concentration of (1*R*,2*S*)-DACH is changed from 0 to 62.5 μM. Error bars represent standard deviations of three independent replicates.

According to the linearly fitted plot shown in Supplementary Fig. 36b, the K_{sv} of *d*-Zn-HOIF NFs towards meso (1*R*,2*S*)-DACH is 5.5×10^3 M^{-1} and just between chiral (1*R*,2*R*)- and (1*S*,2*S*)-DACH. In addition, the LOD is calculated to be 3.2×10^{-6} M.

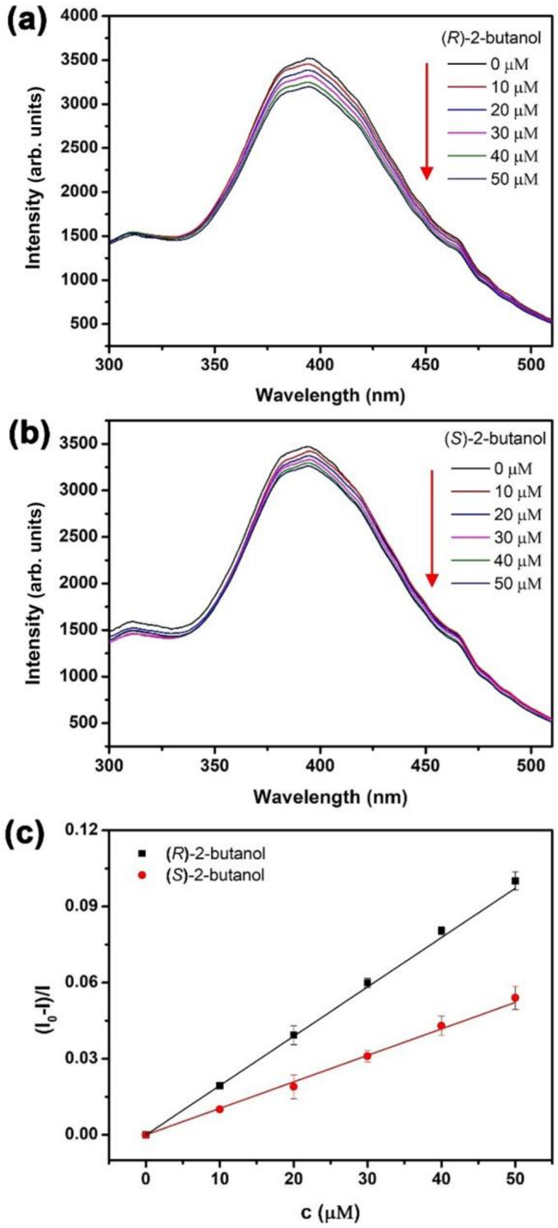

Supplementary Figure 37. Fluorescence quenching spectra of *d*-Zn-HOIF NFs by adding **(a)** (*R*)- and **(b)** (*S*)-2-butanol. **(c)** The corresponding SV plots. The concentration of (*R*)- and (*S*)-2-butanol is changed from 0 to 50 μM. Error bars represent standard deviations of three independent replicates.

According to the plots shown in Supplementary Fig. 37c, the K_{sv} of *d*-Zn-HOIF NFs for (*R*)- and (*S*)-2-butanol is fitted to be 2.0×10^3 and 1.1×10^3 M^{-1}, respectively. As a result, the QR is calculated as 1.8.

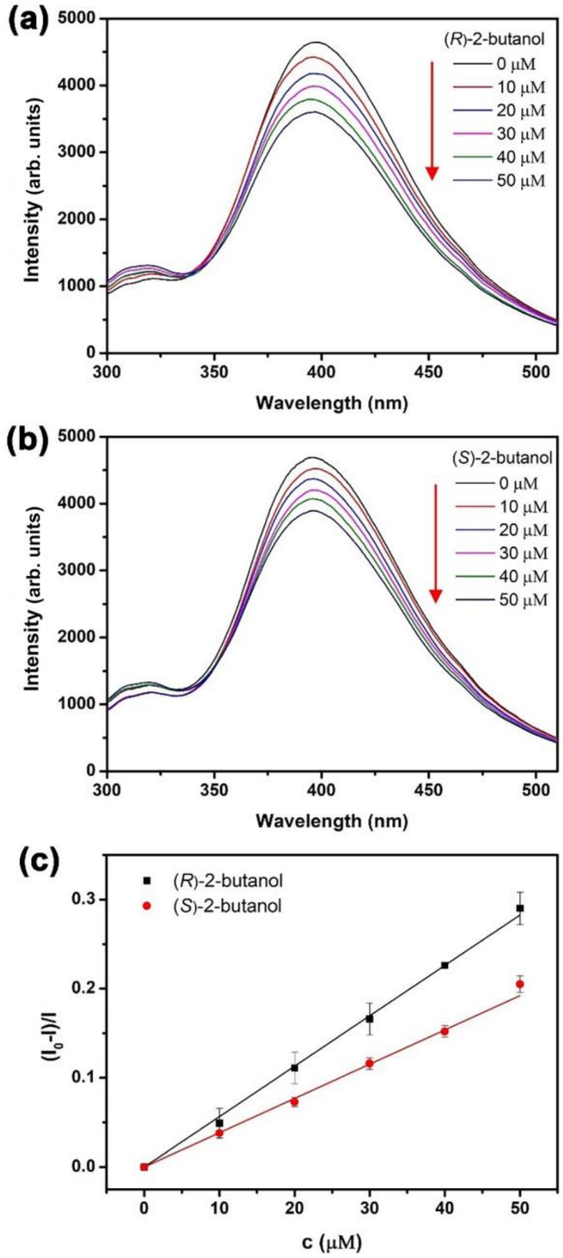

Supplementary Figure 38. Fluorescence quenching spectra of *d*-Zn-HOIF BCs by adding **(a)** (*R*)- and **(b)** (*S*)-2-butanol. **(c)** The corresponding SV plots. The concentration of (*R*)- and (*S*)-2-butanol is changed from 0 to 50 μM. Error bars represent standard deviations of three independent replicates.

According to the plots shown in Supplementary Fig. 38c, the K_{sv} of *d*-Zn-HOIF BCs for (*R*)- and (*S*)-2-butanol is fitted to be 5.7×10^3 and 3.8×10^3 M^{-1}, respectively. As a result, the QR is calculated as 1.5, which is lower than 1.8 of *d*-Zn-HOIF NFs.

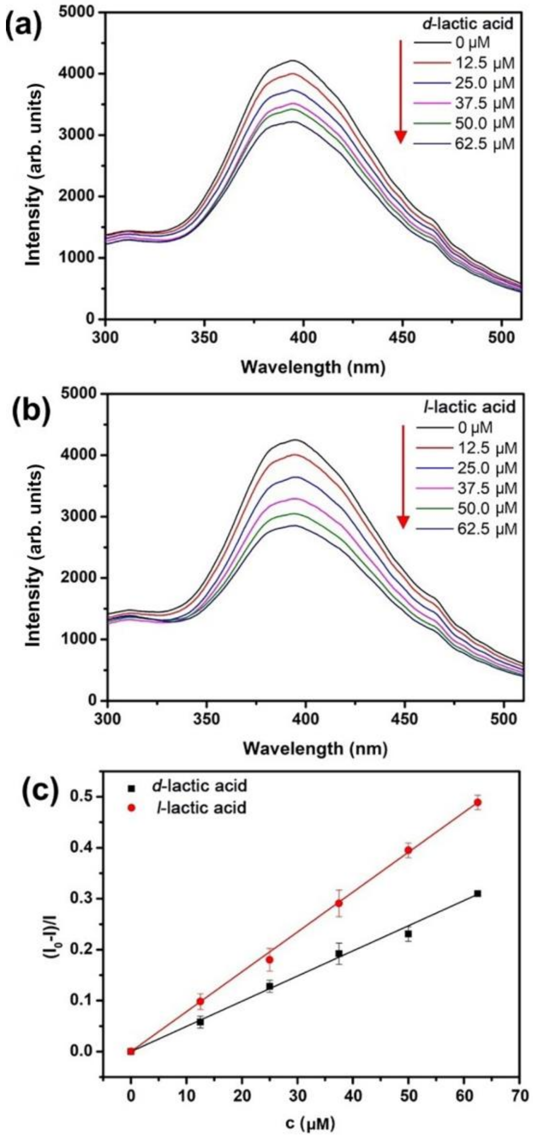

Supplementary Figure 39. Fluorescence quenching spectra of *d*-Zn-HOIF NFs by adding **(a)** (*l*)- and **(b)** (*d*)-lactic acid. **(c)** The corresponding SV plots. The concentration of (*l*)- and (*d*)-lactic acid is changed from 0 to 62.5 μM. Error bars represent standard deviations of three independent replicates.

According to the plots shown in Supplementary Fig. 39c, the K_{sv} of *d*-Zn-HOIF NFs for (*l*)- and (*d*)-lactic acid is fitted to be 7.6×10^3 and 4.8×10^3 M^{-1}, respectively. As a result, the QR is calculated as 1.6.

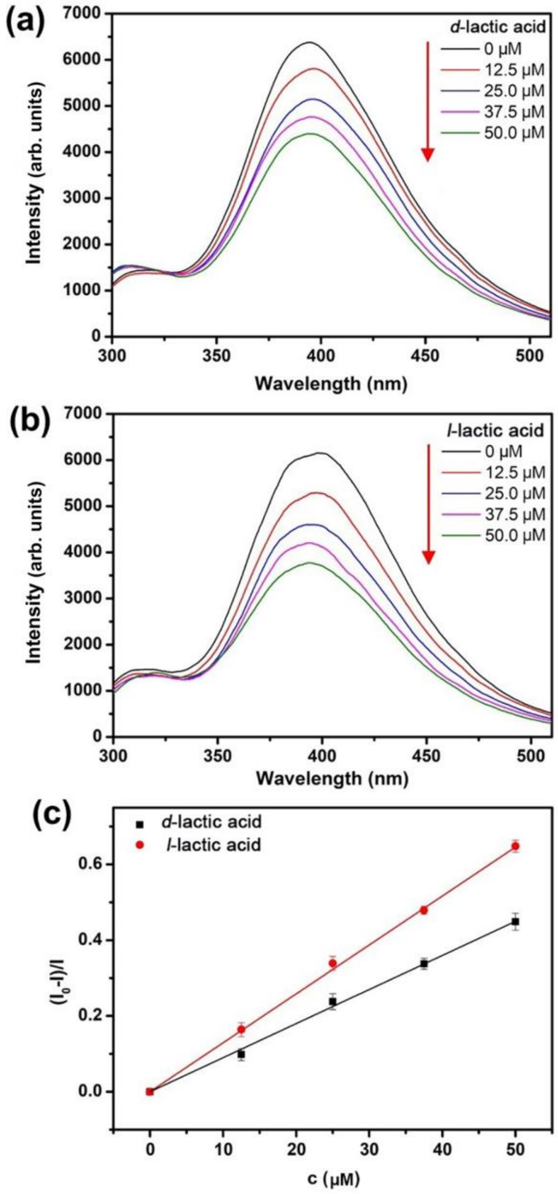

Supplementary Figure 40. Fluorescence quenching spectra of *d*-Zn-HOIF BCs by adding **(a)** (*l*)- and **(b)** (*d*)-lactic acid. **(c)** The corresponding SV plots. The concentration of (*l*)- and (*d*)-lactic acid is changed from 0 to 50 μM. Error bars represent standard deviations of three independent replicates.

According to the plots shown in Supplementary Fig. 40c, the K_{sv} of *d*-Zn-HOIF BCs for (*l*)- and (*d*)-lactic acid is fitted to be 1.3×10^4 and 9.1×10^3 M^{-1}, respectively. As a result, the QR is calculated as 1.4, which is lower than 1.6 of *d*-Zn-HOIF NFs.

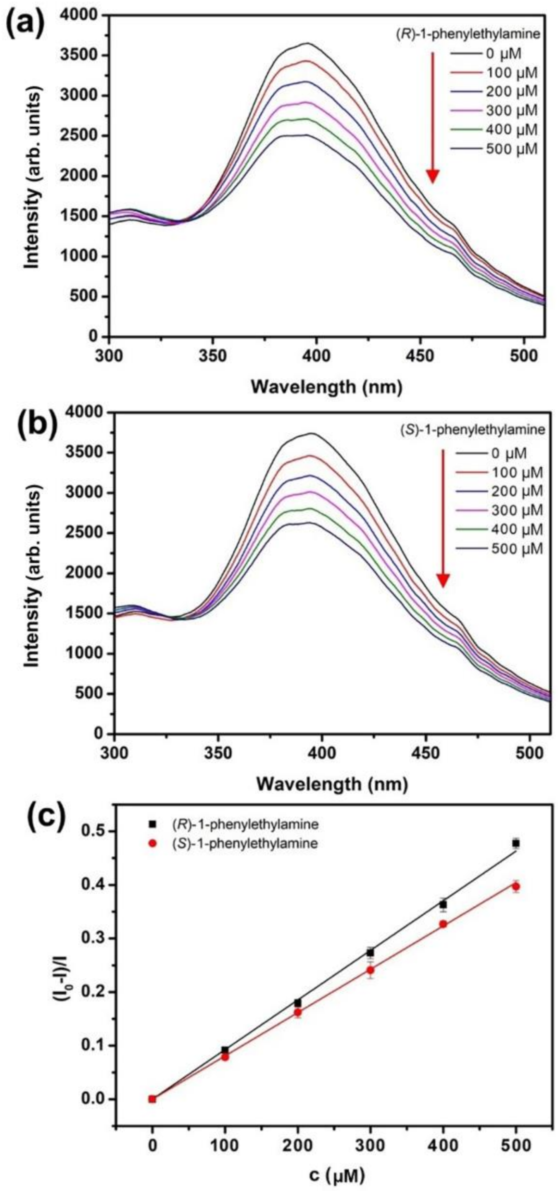

Supplementary Figure 41. Fluorescence quenching spectra of *d*-Zn-HOIF NFs by adding **(a)** (*R*)- and **(b)** (*S*)-1-phenylethylamine. **(c)** The corresponding SV plots. The concentration of (*R*)- and (*S*)-1-phenylethylamine is changed from 0 to 50 mM. Error bars represent standard deviations of three independent replicates.

According to the plots shown in Supplementary Fig. 41c, the K_{sv} of *d*-Zn-HOIF NFs for(*R*)- and (*S*)-1-phenylethylamine is fitted to be 9.3×10^2 and 8.1×10^2 M^{-1}, respectively. As a result, the QR is calculated as 1.1.

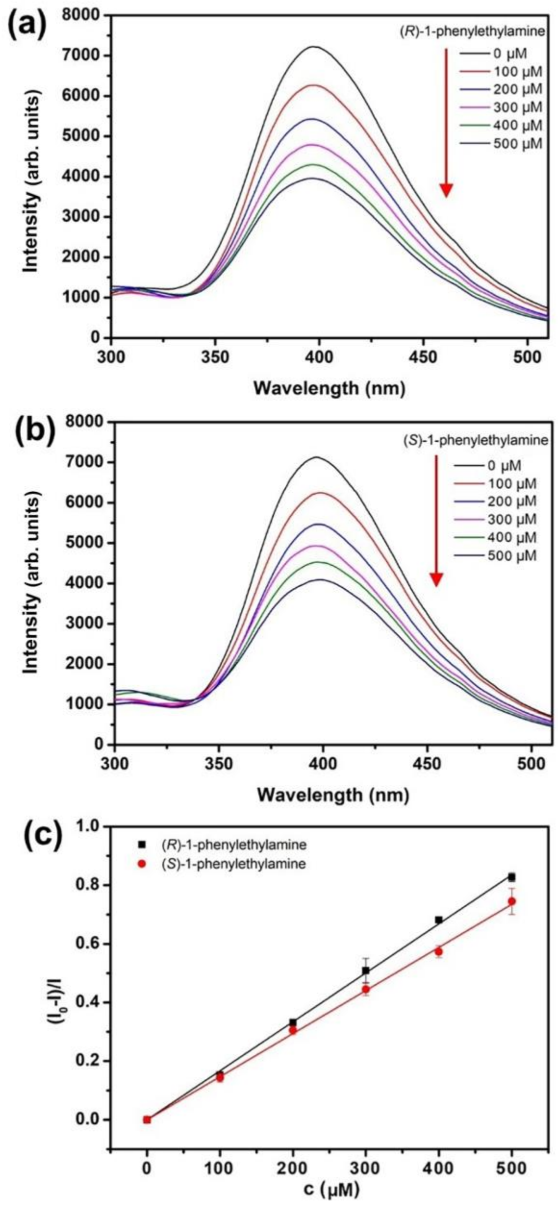

Supplementary Figure 42. Fluorescence quenching spectra of *d*-Zn-HOIF BCs by adding **(a)** (*R*)- and **(b)** (*S*)-1-phenylethylamine. **(c)** The corresponding SV plots. The concentration of (*R*)- and (*S*)-1-phenylethylamine is changed from 0 to 50 mM. Error bars represent standard deviations of three independent replicates.

According to the plots shown in Supplementary Fig. 42c, the K_{sv} of *d*-Zn-HOIF BCs for (*R*)- and (*S*)-1-phenylethylamine is fitted to be 1.7×10^3 and 1.5×10^3 M^{-1}, respectively. As a result, the QR is calculated as 1.1.

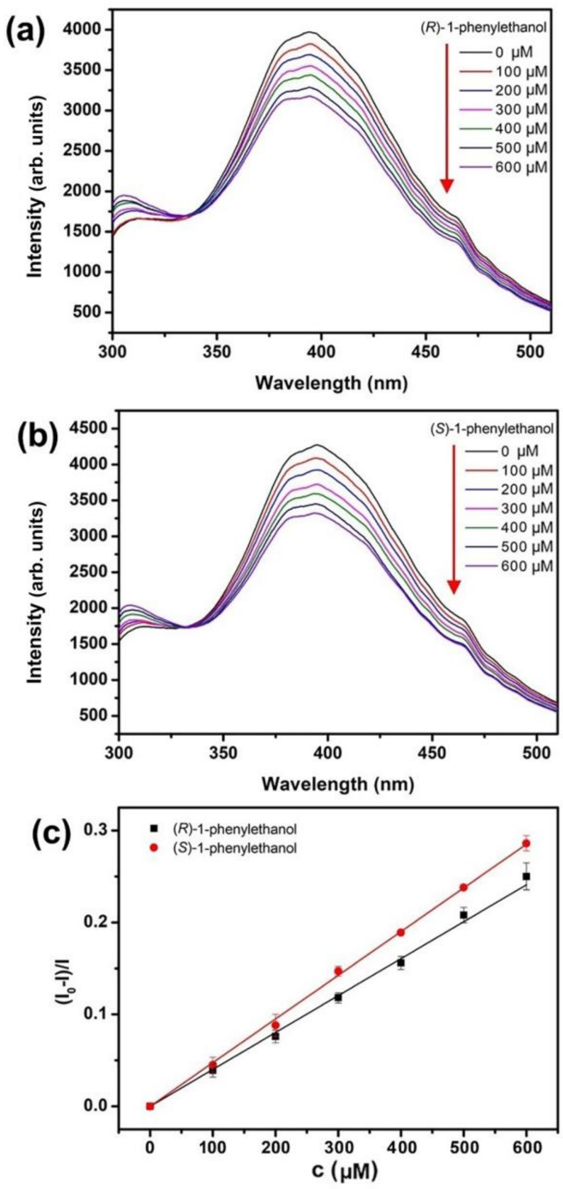

Supplementary Figure 43. Fluorescence quenching spectra of *d*-Zn-HOIF NFs by adding **(a)** (*R*)- and **(b)** (*S*)-1-phenylethanol. **(c)** The corresponding SV plots. The concentration of (*R*)- and (*S*)-1-phenylethanol is changed from 0 to 60 mM. Error bars represent standard deviations of three independent replicates.

According to the plots shown in Supplementary Fig. 43c, the K_{sv} of *d*-Zn-HOIF NFs for (*R*)- and (*S*)-1-phenylethanol is 4.0×10^2 and 4.8×10^2 M^{-1}, respectively. As a result, the QR is calculated as 1.2.

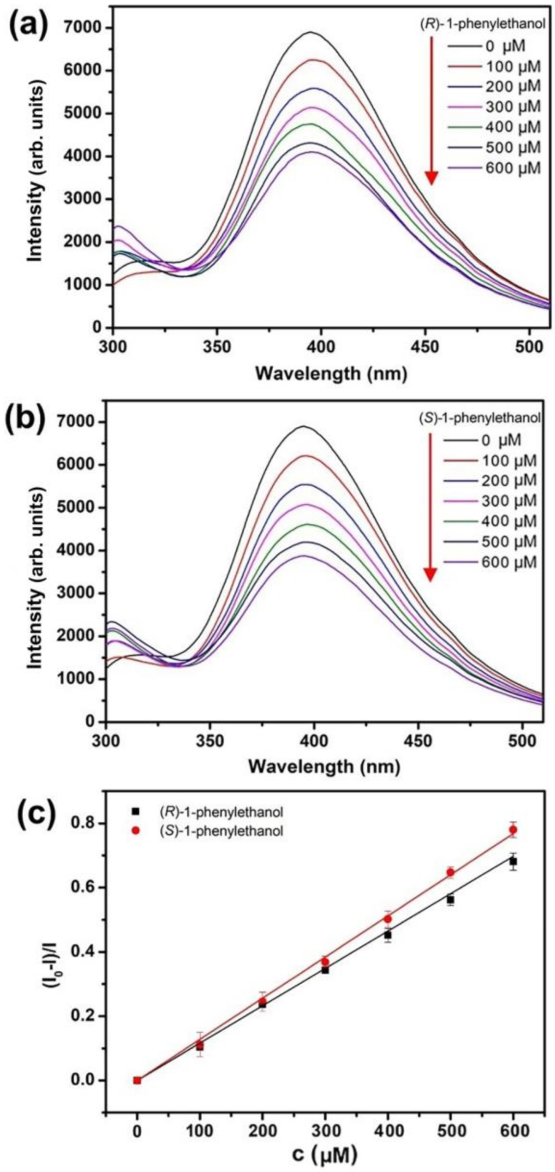

Supplementary Figure 44. Fluorescence quenching spectra of *d*-Zn-HOIF BCs by adding **(a)** (*R*)- and **(b)** (*S*)-1-phenylethanol. **(c)** The corresponding SV plots. The concentration of (*R*)- and (*S*)-1-phenylethanol is changed from 0 mM to 60 mM. Error bars represent standard deviations of three independent replicates.

According to the plots shown in Supplementary Fig. 44c, the K_{sv} of *d*-Zn-HOIF BCs for (*R*)- and (*S*)-1-phenylethanol is fitted to be 1.1×10^3 and 1.3×10^3 M^{-1}, respectively. As a result, the QR is calculated as 1.2.

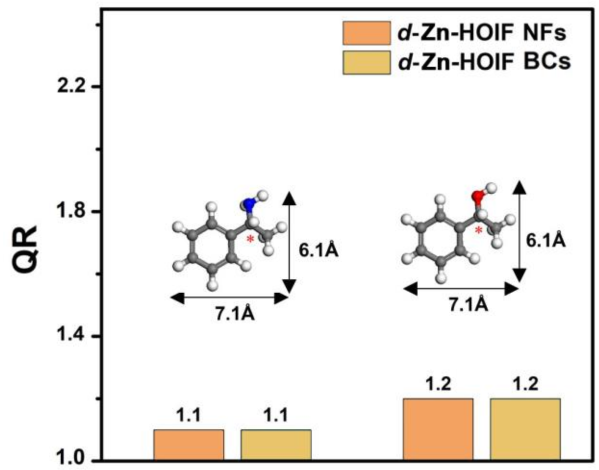

Supplementary Figure 45. QR values of *d*-Zn-HOIF with adding chiral 1-phenylethylamine and 1-phenylethylanol.

As shown in Supplementary Fig. 45, *d*-Zn-HOIF NFs only give a QR value of 1.2 and 1.1 towards aromatic 1-phenylethylanol (7.1 Å × 6.1 Å) and 1-phenylethylamine (7.1 Å × 6.1 Å), respectively. This is attributed to their large steric hindrance, which prevents them from entering the relatively narrow chiral aperture (6.3 Å × 4.8 Å, Supplementary Fig. 46) of *d*-Zn-HOIF NFs.

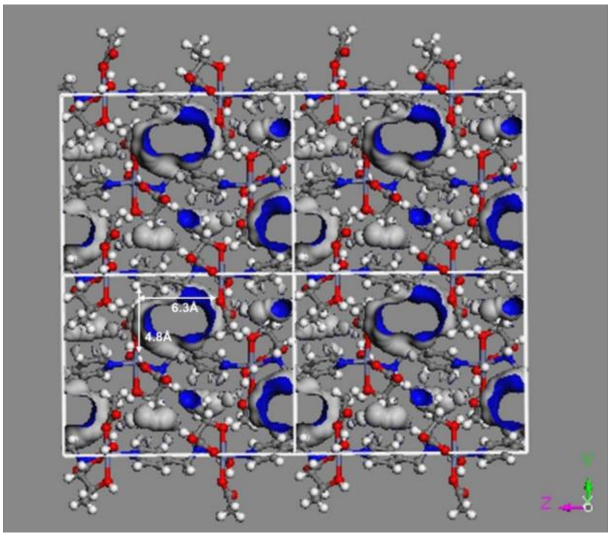

Supplementary Figure 46. Pore texture of *d*-Zn-HOIF based on the well-resolved crystallographic structure.

Supplementary Fig. 46 shows the Connolly volume texture of *d*-Zn-HOIF. The blue and white colors indicate the accessible volumes. The pore size of *d*-Zn-HOIF is estimated to be about 6.3 Å × 4.8 Å.

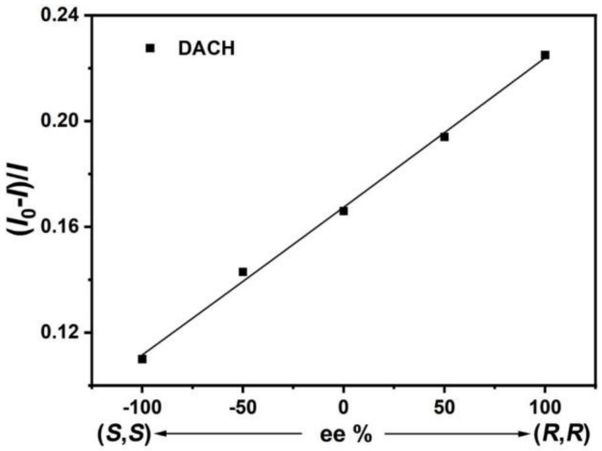

Supplementary Figure 47. SV plot of *d*-Zn-HOIF NFs by adding DACH with different ee values.

Seen from the linearly fitted plot shown in Supplementary Fig. 47, *d*-Zn-HOIF NFs exhibit good recognition for 1,2-DACH of varying ee values, enabling chiral recognitions toward the real chiral substrates with unknown ee values.

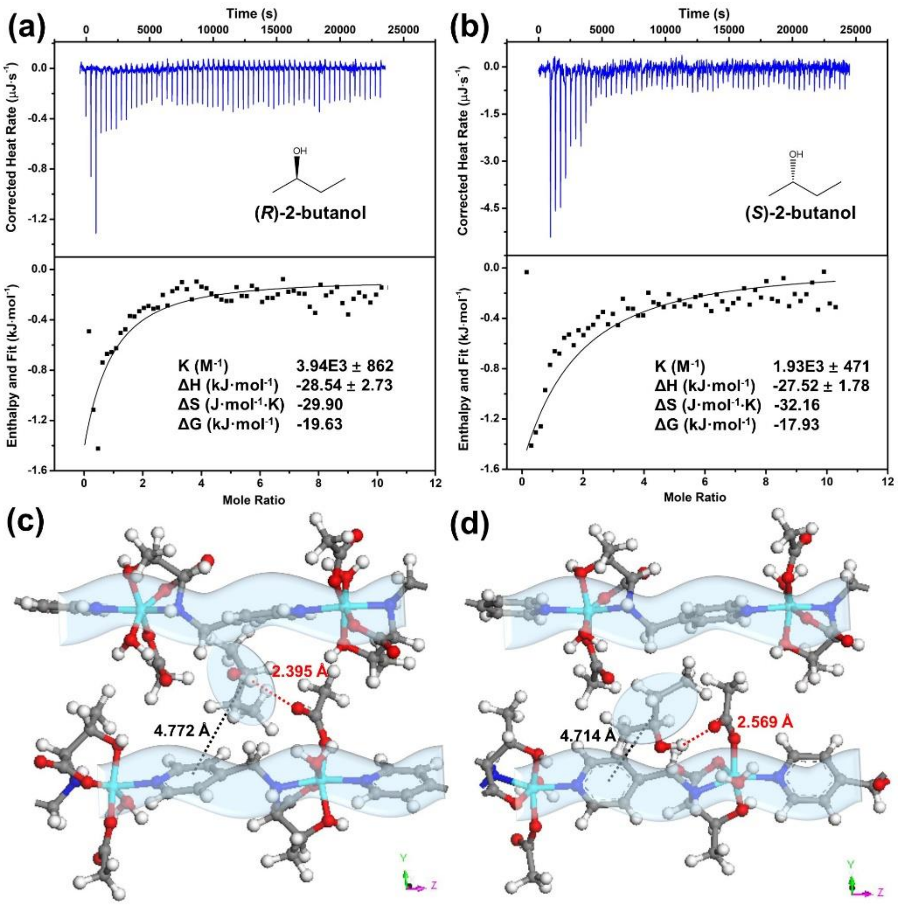

Supplementary Figure 48. ITC curves of *d*-Zn-HOIF NFs with adding **(a)** (*R*)-2-butanol, **(b)** (*S*)-2-butanol. The thermochemical data are obtained by fitting titration curves. The simulated binding interaction between *d*-Zn-HOIF NFs and **(c)** (*R*)-2-butanol, **(d)** (*S*)-2-butanol.

As seen from Supplementary Fig. 48, the association constant (*K*) for (*R*)-2-butanol (3.94×10^3 M^{-1}) determined by ITC is higher than 1.93×10^3 M^{-1} for (*S*)-2-butanol, consistent with the biased affinity derived from fluorescence spectra. Moreover, molecular simulations are performed in order to further verify the ITC experiments. (*R*)-2-Butanol is bound to the dangling acetate oxygen site via donating the chiral hydroxyl group (O-H···O-C, 2.243 Å), stronger than (*S*)-2-butanol (O-H···O-C, 2.454 Å). Moreover, the calculated binding energy for (*R*)-2-butanol is -40.31 kJ·mol^{-1} and larger than -32.26 kJ·mol^{-1} for (*S*)-2-butanol, thus highlighting the stronger binding interaction between (*R*)-2-butanol and *d*-Zn-HOIF NFs.

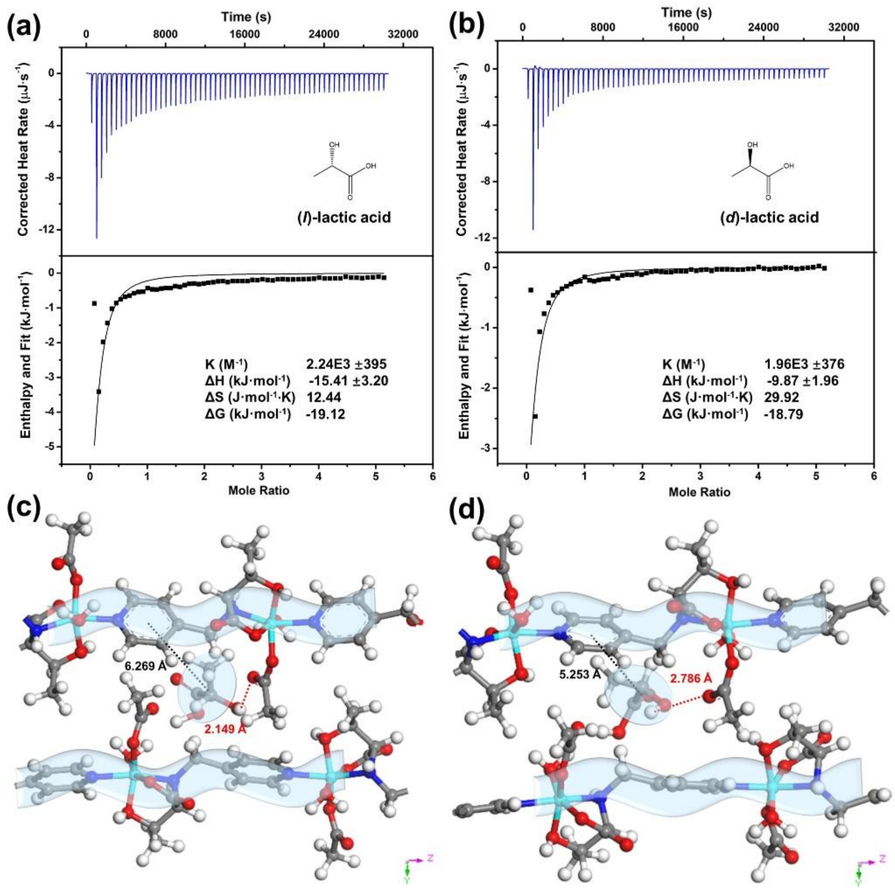

Supplementary Figure 49. ITC curves of *d*-Zn-HOIF NFs with adding **(a)** (*l*)-lactic acid, **(b)** (*d*)-lactic acid. The thermochemical data are obtained by fitting titration curves. The simulated binding interaction between *d*-Zn-HOIF NFs and **(c)** (*l*)-lactic acid, **(d)** (*d*)-lactic acid.

As seen from Supplementary Fig. 49, the association constant (*K*) for (*l*)-lactic acid (2.24×10^3 M^{-1}) determined by ITC is higher than 1.96×10^3 M^{-1} for (*d*)-lactic acid, consistent with the biased affinity derived from fluorescence spectra. Moreover, molecular simulations are performed in order to further verify the ITC experiments. (*l*)-Lactic acid is bound to the dangling acetate oxygen site via donating the chiral hydroxyl group (O-H···O-C, 2.149 Å), stronger than (*d*)-lactic acid (O-H···O-C, 2.786 Å). Moreover, the calculated binding energy for (*l*)-lactic acid is -29.06 kJ·mol^{-1} and larger than -16.12 kJ·mol^{-1} for (*d*)-lactic acid, thus highlighting the stronger binding interaction between (*l*)-lactic acid and *d*-Zn-HOIF NFs.

Supplementary Tables

Supplementary Table 1. The Specific rotation value of *d*-thr and *l*-thr.

Sample	*T* (ºC)	*C* (mg·ml^{-1})	*L* (mm)	*λ* (nm)	Specific rotation (º)
d-thr	30	1	100	589	25.8
l-thr	30	1	100	589	-24.1

Where, *T* is the test temperature, *C* represents the sample concentration in water, *L* stands for the length of sample tube, and *λ* is the test wavelength of light. The *d*-thr and *l*-thr show specific rotation values of +25.8º and -24.1º, respectively.

Supplementary Table 2. Resolved single-crystal data for *d*-Zn-HOIF.

Identification code	*d*-Zn-HOIF
CCDC number	2209408
Empirical formula	$C_{12}H_{18}N_2O_6Zn$
Formula weight	351.65
Temperature/K	169.99(10)
Crystal system	orthorhombic
Space group	$P2_12_12_1$
a/Å	6.14050(10)
b/Å	14.3368(2)
c/Å	18.3682(2)
α/°	90
β/°	90
γ/°	90
Volume/Å^3	1617.05(4)
Z	4
ρ_{calc}g·cm^{-3}	1.444
μ/mm^{-1}	2.362
F(000)	728.0
Crystal size/mm^3	0.14 × 0.1 × 0.08
Radiation	Cu Kα (λ = 1.54184)
2Θ range for data collection/°	7.822 to 147.62
Index ranges	$-7 \leqslant h \leqslant 5, -17 \leqslant k \leqslant 17, -22 \leqslant l \leqslant 22$
Reflections collected	12426
Independent reflections	3212 [R_{int} = 0.0545, R_{sigma} = 0.0469]
Data/restraints/parameters	3212/0/197
Goodness-of-fit on F^2	1.010

Final R indexes [I>=2σ (I)]	R_1 = 0.0341, wR_2 = 0.0842
Final R indexes [all data]	R_1 = 0.0397, wR_2 = 0.0857
Largest diff. peak/hole / e Å^{-3}	0.33/-0.37
Flack/Hooft parameter	-0.016(19)/0.007(16)

Supplementary Table 3. Fractional atomic coordinates (×10^4) and equivalent isotropic displacement parameters (Å^2×10^3) for *d*-Zn-HOIF. U_{eq} is defined as 1/3 of the trace of the orthogonalized U_{IJ} tensor.

Atom	*x*	*y*	*z*	U(eq)
Zn1	2962.3(8)	4871.4(3)	1884.0(2)	18.23(14)
O1	6806(6)	4138(2)	5554.9(16)	35.5(7)
O2	5174(4)	4867(2)	6479.4(14)	23.0(6)
O3	1767(6)	3527.4(19)	6870.7(15)	27.0(6)
O4	2102(6)	3479.8(18)	1822.5(17)	31.6(6)
O5	5022(6)	2758(2)	2267(2)	38.6(8)
$O6_w$	6211(5)	4542(2)	2125.4(14)	23.6(6)
N1	2331(5)	4970(2)	3004.7(15)	22.3(6)
N2	1292(5)	4695(2)	5794.9(15)	16.5(6)
C1	566(7)	4671(3)	3349(2)	28.0(9)
C2	227(7)	4803(3)	4090(2)	29.8(9)
C3	1787(7)	5269(3)	4495.9(19)	24.4(8)
C4	3629(9)	5569(4)	4145(2)	36.2(11)
C5	3849(9)	5410(4)	3405(2)	35.9(11)
C6	1511(8)	5494(3)	5297(2)	30.1(11)
C7	5197(7)	4353(3)	5911(2)	20.8(8)
C8	2940(7)	3966(2)	5690.2(18)	17.1(7)
C9	2379(7)	3130(3)	6184(2)	22.5(9)
C10	636(9)	2504(3)	5875(2)	32.6(10)
C11	1644(9)	1928(3)	2221(3)	45.3(13)
C12	3022(9)	2785(3)	2101(2)	27.4(8)

Supplementary Table 4. Hydrogen atom coordinates (Å×10^4) and isotropic displacement parameters (Å^2×10^3) for *d*-Zn-HOIF.

Atom	*x*	*y*	*z*	U(eq)
H3	1250(100)	3160(40)	7130(30)	32(15)
H6A	7025.8	4709.36	1773.23	35
H6B	6358.67	3952.02	2144.49	35
H2	-208.66	4425.42	5768.4	20
H1	-516.53	4350.86	3076.21	34
H2A	-1060.66	4575.86	4315.5	36
H4	4742.18	5884.09	4406.65	43
H5	5134.78	5621.16	3168.89	43
H6C	2781.1	5867.99	5455.08	36
H6D	202.7	5891.82	5351.67	36
H8	2964.09	3763.58	5169.19	21
H9	3728.88	2751.72	6257.19	27
H10A	356.97	1989.28	6213.68	49
H10B	1124.12	2251.76	5406.63	49
H10C	-704.76	2862.97	5803.73	49
H11A	180.37	2114.68	2374.81	68
H11B	2309.64	1538.5	2598.74	68
H11C	1548.31	1572.34	1765.91	68

Supplementary Table 5. Bond lengths (Å) for *d*-Zn-HOIF.

Atom	Atom	Length/Å	Atom	Atom	Length/Å
Zn1	O2[1]	2.098(3)	N1	C5	1.344(6)
Zn1	O3[1]	2.302(3)	N2	C6	1.472(5)
Zn1	O4	2.067(3)	N2	C8	1.468(5)
Zn1	$O6_w$	2.097(3)	C1	C2	1.390(5)
Zn1	N1	2.099(3)	C2	C3	1.386(6)
Zn1	N2[1]	2.144(3)	C3	C4	1.371(7)
O1	C7	1.225(5)	C3	C6	1.516(5)
O2	C7	1.277(5)	C4	C5	1.385(6)
O3	C9	1.433(4)	C7	C8	1.547(6)
O4	C12	1.254(5)	C8	C9	1.541(5)
O5	C12	1.266(6)	C9	C10	1.508(6)
N1	C1	1.326(5)	C11	C12	1.508(6)

[1]1/2-X,1-Y,-1/2+Z

Supplementary Table 6. Bond angles (°) for *d*-Zn-HOIF.

Atom	Atom	Atom	Angle/°	Atom	Atom	Atom	Angle/°
O2^{1}	Zn1	O3^{1}	83.36(12)	C8	N2	Zn1^{2}	100.5(2)
O2^{1}	Zn1	N2^{1}	79.27(11)	C8	N2	C6	114.2(3)
O4	Zn1	O2^{1}	85.32(13)	N1	C1	C2	123.0(4)
O4	Zn1	O3^{1}	168.68(14)	C3	C2	C1	119.3(4)
O4	Zn1	O6$_{w}$	92.14(13)	C2	C3	C6	123.2(4)
O4	Zn1	N1	94.10(13)	C4	C3	C2	117.9(4)
O4	Zn1	N2^{1}	106.48(12)	C4	C3	C6	118.8(4)
O6$_{w}$	Zn1	O2^{1}	171.18(11)	C3	C4	C5	119.4(4)
O6$_{w}$	Zn1	O3^{1}	99.09(12)	N1	C5	C4	123.1(4)
O6$_{w}$	Zn1	N1	89.06(12)	N2	C6	C3	116.6(3)
O6$_{w}$	Zn1	N2^{1}	93.40(11)	O1	C7	O2	126.2(4)
N1	Zn1	O2^{1}	99.54(11)	O1	C7	C8	119.5(3)
N1	Zn1	O3^{1}	87.51(12)	O2	C7	C8	114.3(3)
N1	Zn1	N2^{1}	159.15(12)	N2	C8	C7	109.1(3)
N2^{1}	Zn1	O3^{1}	71.65(11)	N2	C8	C9	108.8(3)
C7	O2	Zn1^{2}	113.7(2)	C9	C8	C7	109.0(3)
C9	O3	Zn1^{2}	112.7(2)	O3	C9	C8	105.5(3)
C12	O4	Zn1	129.0(3)	O3	C9	C10	112.4(3)
C1	N1	Zn1	126.6(3)	C10	C9	C8	113.6(3)
C1	N1	C5	117.3(3)	O4	C12	O5	124.0(4)
C5	N1	Zn1	116.1(3)	O4	C12	C11	117.0(5)
C6	N2	Zn1^{2}	109.5(2)	O5	C12	C11	119.0(4)

^{1}1/2-X,1-Y,-1/2+Z;

^{2}1/2-X,1-Y,1/2+Z

Supplementary Table 7. Hydrogen bonds for *d*-Zn-HOIF [Å and °].

D[1]-H...X[2]	*d*(D-H)	*d*(H...X)	*d*(D...X)	<(DHX)
N2-H2...O1	1.000(4)	1.919(4)	2.902(5)	166.55(19)
$O6_w$ -H6A...O2	0.852(3)	1.902(3)	2.656(4)	146.7(3)
$O6_w$ -H6B...O5	0.851(3)	1.912(4)	2.673(5)	148.1(3)
O3-H3...O5	0.780(6)	1.880(6)	2.656(5)	178.0(6)

“D” stands for hydrogen-donor atom;

“X” stands for hydrogen-acceptor atom.

Supplementary Table 8. Resolved single-crystal data for *l*-Zn-HOIF.

Identification code	*l*-Zn-HOIF
CCDC number	2209409
Empirical formula	$C_{12}H_{18}N_2O_6Zn$
Formula weight	351.65
Temperature/K	169.99(10)
Crystal system	orthorhombic
Space group	$P2_12_12_1$
a/Å	6.1394(2)
b/Å	14.4103(5)
c/Å	18.3669(6)
α/°	90
β/°	90
γ/°	90
*V*olume/Å^3	1624.93(10)
Z	4
ρ_{calc}g·cm^{-3}	1.437
μ/mm^{-1}	2.351
F(000)	728.0
Crystal size/mm^3	0.14 × 0.11 × 0.09
Radiation	Cu Kα (λ = 1.54184)
2Θ range for data collection/°	7.798 to 147.514
Index ranges	$-7 \leqslant h \leqslant 6, -17 \leqslant k \leqslant 17, -11 \leqslant l \leqslant 22$
Reflections collected	4302
Independent reflections	2759 [R_{int} = 0.0358, R_{sigma} = 0.0567]
Data/restraints/parameters	2759/3/200
Goodness-of-fit on F^2	1.024

Final R indexes [I>=2σ (I)]	R_1 = 0.0383, wR_2 = 0.0960
Final R indexes [all data]	R_1 = 0.0435, wR_2 = 0.0996
Largest diff. peak/hole / e $Å^{-3}$	0.49/-0.58
Flack parameter	0.04(4)

Supplementary Table 9. Fractional atomic coordinates (×10^4) and equivalent isotropic displacement parameters (Å^2×10^3) for *l*-Zn-HOIF. U_{eq} is defined as 1/3 of the trace of the orthogonalized U_{IJ} tensor.

Atom	*x*	*y*	*z*	U(eq)
Zn1	2081.2(9)	4940.4(4)	1941.9(3)	15.64(16)
O1	3214(6)	3479(2)	6885.5(18)	24.0(7)
O2	-213(5)	4826(2)	6524.7(17)	18.8(7)
O3	-1829(6)	4137(3)	5579(2)	29.2(9)
$O4_w$	-1158(5)	4615(2)	2189.3(18)	20.8(7)
O5	2968(6)	3550(2)	1906.3(19)	23.0(7)
O6	-46(7)	2824(3)	2269(2)	32.6(9)
N1	3679(6)	4690(3)	5838(2)	14.5(8)
N2	2718(6)	5069(3)	3062.1(19)	20.8(8)
C1	4346(9)	2505(4)	5860(3)	29.6(13)
C2	2562(8)	3103(3)	6157(3)	19.5(11)
C3	2033(8)	3968(3)	5710(2)	15.4(9)
C4	-220(7)	4344(3)	5937(3)	15.6(9)
C5	3469(9)	5520(3)	5360(2)	22.3(11)
C6	3240(8)	5330(3)	4555(2)	21.0(10)
C7	1408(9)	5649(4)	4202(3)	31.3(13)
C8	1196(11)	5499(4)	3462(3)	35.1(14)
C9	4475(8)	4752(4)	3406(3)	23.0(11)
C10	4792(8)	4865(4)	4148(3)	28.5(12)
C11	1960(9)	2841(3)	2146(3)	22.3(10)
C12	3290(9)	1978(4)	2257(4)	38.5(15)

Supplementary Table 10. Hydrogen atom coordinates (Å×10^4) and isotropic displacement parameters (Å^2×10^3) for *l*-Zn-HOIF.

Atom	*x*	*y*	*z*	U(eq)
H1	3860(100)	4150(20)	7141(16)	36
H4A	-1369.01	4020.23	2167.53	31
H4B	-2028.65	4833.2	1858.38	31
H1A	5070(90)	4410(30)	5780(30)	16(13)
H1B	5629.57	2873.18	5787.09	44
H1C	3893.48	2241.76	5404.56	44
H1D	4658.3	2015.1	6199.25	44
H2	1237.99	2729.23	6212.66	23
H3	2010.39	3807.76	5191.98	18
H5A	4740.72	5908.77	5431.75	27
H5B	2208.64	5872.73	5517.63	27
H7	327.96	5962.58	4458.33	38
H8	-60.48	5706.19	3230.52	42
H9	5534.07	4442.8	3138.03	28
H10	6036.07	4629.54	4370.15	34
H12A	3209.43	1598.32	1828.78	58
H12B	2731.9	1639.44	2666.63	58
H12C	4779.66	2145.77	2346.99	58

Supplementary Table 11. Bond lengths (Å) for *l*-Zn-HOIF.

Atom	Atom	Length/Å	Atom	Atom	Length/Å
Zn1	O1[1]	2.287(3)	N1	C5	1.490(6)
Zn1	O2[1]	2.097(3)	N2	C8	1.340(7)
Zn1	$O4_w$	2.093(3)	N2	C9	1.331(6)
Zn1	O5	2.078(3)	C1	C2	1.497(6)
Zn1	N1[1]	2.148(4)	C2	C3	1.528(6)
Zn1	N2	2.102(4)	C3	C4	1.543(6)
O1	C2	1.498(5)	C5	C6	1.509(6)
O2	C4	1.283(6)	C6	C7	1.377(7)
O3	C4	1.223(6)	C6	C10	1.385(7)
O5	C11	1.273(6)	C7	C8	1.383(7)
O6	C11	1.252(7)	C9	C10	1.385(7)
N1	C3	1.468(6)	C11	C12	1.502(7)

[1]1/2-X,1-Y,-1/2+Z

Supplementary Table 12. Bond angles (˚) for *l*-Zn-HOIF.

Atom	Atom	Atom	Angle/˚	Atom	Atom	Atom	Angle/˚
O2^{1}	Zn1	O1^{1}	84.04(13)	C9	N2	Zn1	125.9(3)
O2^{1}	Zn1	N1^{1}	79.31(13)	C9	N2	C8	117.6(4)
O2^{1}	Zn1	N2	99.95(13)	O1	C2	C3	103.9(3)
O4$_{w}$	Zn1	O1^{1}	99.06(13)	C1	C2	O1	109.8(4)
O4$_{w}$	Zn1	O2^{1}	170.57(13)	C1	C2	C3	115.5(4)
O4$_{w}$	Zn1	N1^{1}	93.10(14)	N1	C3	C2	110.2(4)
O4$_{w}$	Zn1	N2	89.08(14)	N1	C3	C4	109.0(4)
O5	Zn1	O1^{1}	168.49(15)	C2	C3	C4	109.4(4)
O5	Zn1	O2^{1}	84.45(13)	O2	C4	C3	114.4(4)
O5	Zn1	O4$_{w}$	92.28(14)	O3	C4	O2	125.9(4)
O5	Zn1	N1^{1}	105.47(14)	O3	C4	C3	119.6(4)
O5	Zn1	N2	93.84(14)	N1	C5	C6	116.0(4)
N1^{1}	Zn1	O1^{1}	72.11(13)	C7	C6	C5	118.4(5)
N2	Zn1	O1^{1}	88.36(14)	C7	C6	C10	118.0(5)
N2	Zn1	N1^{1}	160.46(15)	C10	C6	C5	123.6(5)
C2	O1	Zn1^{2}	112.3(2)	C6	C7	C8	119.2(5)
C4	O2	Zn1^{2}	113.4(3)	N2	C8	C7	123.1(5)
C11	O5	Zn1	129.4(3)	N2	C9	C10	122.8(5)
C3	N1	Zn1^{2}	100.2(3)	C6	C10	C9	119.4(5)
C3	N1	C5	114.5(4)	O5	C11	C12	116.5(5)
C5	N1	Zn1^{2}	109.8(3)	O6	C11	O5	123.8(5)
C8	N2	Zn1	116.6(3)	O6	C11	C12	119.6(5)

^{1}1/2-X,1-Y,-1/2+Z;

^{2}1/2-X,1-Y,1/2+Z

Supplementary Table 13. Hydrogen bonds for *l*-Zn-HOIF [Å and °].

D^1-H...X^2	*d*(D-H)	*d*(H...X)	*d*(D...X)	<(DHX)
N1-H1A...O3	0.950(6)	1.980(6)	2.910(6)	166.0(5)
$O4_w$-H4B...O2	0.868(4)	1.867(4)	2.665(5)	152.1(3)
$O4_w$-H4A...O6	0.868(3)	1.915(5)	2.674(6)	145.2(3)
O1-H1...O6	1.150(4)	3.120(4)	2.661(6)	56.4(15)

“D” stands for hydrogen-donor atom;

“X” stands for hydrogen-acceptor atom.

Supplementary Table 14. The refined cell parameters based on Pawley refinement method.

Sample	Crystal system	Space group	*a* (Å)	*b* (Å)	*c* (Å)	α (°)	β (°)	λ (°)	*r* (Å)
d-Co-HOIF	Orthorhombic	$P2_12_12_1$	6.14	14.17	18.40	90	90	90	0.72
l-Co-HOIF	Orthorhombic	$P2_12_12_1$	6.14	14.17	18.40	90	90	90	0.72
d-Ni-HOIF	Orthorhombic	$P2_12_12_1$	6.11	13.97	18.23	90	90	90	0.69
l-Ni-HOIF	Orthorhombic	$P2_12_12_1$	6.13	14.02	18.32	90	90	90	0.69
d-Zn-HOIF	Orthorhombic	$P2_12_12_1$	6.14	14.34	18.36	90	90	90	0.74
l-Zn-HOIF	Orthorhombic	$P2_12_12_1$	6.14	14.41	18.37	90	90	90	0.74

In addition, we have carefully analyzed the PXRD results of both *d*(*l*)-Ni-HOIFs and *d*(*l*)-Co-HOIFs by carrying out PXRD refinements (Supplementary Fig. 13) in order to offer corresponding cell parameters. As results collected in Supplementary Table 14, the referee can clearly see the smaller cell parameters of *d*(*l*)-Ni-HOIFs especially along the *b*-axis and *c*-axis, due to the smaller ionic radius (0.69 Å) of Ni^{2+} compared with Co^{2+} (0.72 Å) and Zn^{2+} (0.74 Å).

Supplementary Table 15. Anisotropic factors (g_{lum}) comparison between Zn-HOIF NFs with recently reported chiral porous frameworks.

No.	Material type	Composition	g_{lum}	Wavelength (nm)	Ref.
1	*Δ*/*Λ*-1·2G$_1$ (Hexahedral Pd_6L_{12} cages) (MOC)	Pd_6L_{12} (L = Penta(ethylene glycol)di-p-toluenesulfonate and enantiopure 5,5',6,6'-tetramethyl-3,30-di(pyridin-4-yl)-biphenyl-2,20-diol)	1.0×10^{-3}	550	***Chem*** 2017, 7, 2771.[9]
2	*Δ*/*Λ*-TpTab (Chiral COF)	TpTab (Tab = 1,3,5-Tris(4-aminophenyl)benzene, TP = 1,3,5-triformylphloroglucinol)	---	540 (CPL not available)	***Nat. Comm.*** 2018, 9, 1294.[10]
3	*R*/*S*-ZIF (ZIF-8 variant) (ZIF)	$Zn_3(L)_6$ (L = 2-(1H-imidazol-4-yl)-N-(2'-((4-(10-phenylanthracen-9-yl)phenyl)amino)-[1,1'-binaphthalen]-2-yl)acetamide)	7.0×10^{-4}	300	***Angew. Chem. Int. Ed.*** 2019, 131, 5032.[11]
4	Zn-MOF-Tb (Chiral MOF)	$([(CH_3)_2NH_2]_{1/2}[Zn_2(adenine)(TATAB)O_{1/4}]$ (H_3TATAB = 4,4',4''-s-triazine-1,3,5-triyltri-p-aminobenzoic acid)	---	544 (CPL not available)	***Nat. Comm.*** 2019, 10, 5117.[12]
5	[Zn(*d*/*l*-*l*$_{Cl}$)(Cl)]$(H_2O)_2$ (MOF)	[Zn(*d*/*l*-*l*$_{Cl}$)(Cl)]$(H_2O)_2$ (*d*/*l*-*l*$_{Cl}$ = *N*-(4-pyridylmethyl)-*d*/*l*-valine)	1.5×10^{-3}	420	***Adv. Mater.*** 2020, 32, e2002914.[8]
6	*d*/*l*-ZIF ⊃ DCM (ZIF-8 variant) (ZIF)	$Zn_3[(Hmim)_x$(*d*/*l*-His)$_{1-x}]_6$ ⊃ DCM (Hmim = 2-methylimidazole, *d*/*l*-His, DCM = 4-(dicyanomethylene)-2-methyl-6-(4-dimethylaminostyryl)-4H-pyran)	1.2×10^{-3}	575	***Research*** 2020, 2020, 6452123.[13]
7	*P*/*M*-TbBTC (MOF)	TbBTC (H_3BTC = 1,3,5-benzenetricarboxylic acid)	1.3×10^{-3}	540	***Chem. Sci.*** 2020, 11, 9154.[14]
8	*P*/*M*-2 (MOF with eta topology)	$Zn_2Br_2(L_1)$ (L_1 = *N*,*N*'-(1,2-cyclohexane-1,2-diyl)bis(1-(2-methyl-1H-	1.0×10^{-3}	525	***Adv. Opt. Mater.*** 2021, 9, 2002096.[15]

		imidazole-4-carboxaldehyde))			
9	Axially chiral COF	$(C)_2(L)_3$ (C = 1,3,5-tris(4,4,5,5-tetramethyl-1,3,2-dioxaborolan-2-yl)benzene, L = 4,4''-dibromo-1,1':4',1''-terphenyl)	2.5×10^{-4}	326	***Chem. Commun.*** 2021, 57, 7681.[16]
10	*d*/*l*-Eu(BTC)(H_2O) (MOF)	Eu(BTC)(H_2O) (H_3BTC = 1,3,5-benzenetricarboxylic acid)	4.0×10^{-4}	620	***Adv. Mater.*** 2022, 34, 2109496.[17]
11	*P*/*M*-[(CuI)-(Hptdp)]$_4$·H_2O (MOF)	[(CuI)(Hptdp)]$_4$·H_2O (Hptdp = 4-(pyridin-4'-ylthio)-3,5-dimethyl-1H-pyrazole)	6.0×10^{-4}	520	***Angew. Chem. Int. Ed.*** 2022, 61, e202201590.[18]
12	*d*/*l*-Zn-CMOF (MOF)	Zn(*d*/*l*-cam)$_2$(TPB)$_{1/2}$ (*d*/*l*-cam = *d*/*l*-camphorates, TPB = 1,2,4,5-tetra(pyridin-4-yl)benzene)	8.0×10^{-4}	410	***ACS Appl. Mater. Interfaces*** 2022, 14, 16435.[19]
13	BIT-309 (MOF)	Eu_3(DTABPIPA)$_4$(NO_3)(H_2O)$_9$ (DTABPIPA = 3',5'-di(1H-1,2,4-triazol-1-yl)-[1,1'-biphenyl]-3,5-dicarboxylic)	1.1×10^{-3}	620	***Chem. Eur. J.*** 2023, 29, e202203534.[20]
14	*R*/*S*-1M (Micellar aggregate)	3-phenylperylene complex and hexadecyltrimethyl ammonium bromide	1.5×10^{-3}	470	***Nat. Comm.*** 2023, 14, 81.[21]
15	*d*(*l*)-Zn-HOIF NFs	Zn(*d*/*l*-thr)(CH_3COO)H_2O (*d*/*l*-thr = *N*-(4-pyridylmethyl)-*d*/*l*-threonine)	1.7×10^{-3}	405	**This work**

Supplementary Table 16. Summary of fluorescence based enantioselective recognition towards different chiral molecules by *d*-Zn-HOIF NFs.

HOIFs	Chiral enantiomers	K_{sv} (M^{-1})	LOD (M)	QR
d-Zn-HOIF NFs	(1*R*,2*R*)-DACH	7.3×10^3	2.8×10^{-6}	2.0
	(1*S*,2*S*)-DACH	3.6×10^3	5.1×10^{-6}	
	(*R*)-2-butanol	2.0×10^3	2.3×10^{-6}	1.8
	(*S*)-2-butanol	1.1×10^3	4.3×10^{-6}	
	l-lactic acid	7.6×10^3	3.9×10^{-6}	1.6
	d-lactic acid	4.8×10^3	4.8×10^{-6}	
	(*R*)-1-phenylethylamine	9.3×10^2	2.4×10^{-5}	1.1
	(*S*)-1-phenylethylamine	8.1×10^2	1.4×10^{-5}	
	(*R*)-1-phenylethanol	4.0×10^2	2.9×10^{-5}	1.2
	(*S*)-1-phenylethanol	4.4×10^2	2.7×10^{-5}	

The limit of detection (LOD) is defined as follows[22]:

$$LOD = 3\sigma/K_{sv}$$

where K_{sv} is the quenching constant and σ is the standard deviation. Specifically, σ can be calculated following:

$$\sigma = \sqrt{\sum_{mi=1}^{n} (mi - mf)^2/n}$$

where m_i is experimentally obtained $(I_0\text{-}I)/I$, m_f is the linearly fitted $(I_0\text{-}I)/I$ value according to the "Stern-Volmer" equation, and n is the number of sampling points.

Supplementary Table 17. Summary of ITC, fluorescence and molecular simulation data for *d*-Zn-HOIFs NFs.

Chiral enantiomer	ITC K (M^{-1})	ΔH ($kJ\cdot mol^{-1}$)	$T\Delta S$ ($kJ\cdot mol^{-1}$)	ΔG ($kJ\cdot mol^{-1}$)	FL K_{SV} (M^{-1})	Simulated ΔE ($kJ\cdot mol^{-1}$)
(1*R*,2*R*)-DACH	8.1×10^3	-46.00	-23.69	-22.31	7.3×10^3	-48.45
(1*S*,2*S*)-DACH	6.3×10^3	-39.18	-17.51	-21.67	3.6×10^3	-43.32
(*R*)-2-butanol	3.9×10^3	-28.54	-8.91	-19.63	2.0×10^3	-40.31
(*S*)-2-butanol	1.9×10^3	-27.52	-9.59	-17.93	1.1×10^3	-32.26
l-lactic acid	2.2×10^3	-15.41	3.71	-19.12	7.6×10^3	-29.06
d-lactic acid	2.0×10^3	-9.87	8.92	-18.79	4.8×10^3	-16.12

Supplementary References

1. Momma K., Izumi F., Vesta 3 for three-dimensional visualization of crystal, volumetric and morphology data. *J. Appl. Crystallogr.* **44**, 1272-1276 (2011).
2. Putz H., Brandenburg K., Diamond-crystal and molecular structure visualization. *GbR. Kreuzherrenstr* **102**, 53227 (2006).
3. Chen H., Liu L., Qian K., Liu H., Wang Z., Gao F., Qu C., Dai W., Lin D., Chen K., Bioinspired large stokes shift small molecular dyes for biomedical fluorescence imaging. *Sci. Adv.* **8**, eabo3289 (2022).
4. Jin J. L., Li H. B., Geng Y., Wu Y., Duan Y. A., Su Z. M., Theoretical insight into the origin of large stokes shift and photophysical properties of anilido-pyridine boron difluoride dyes. *ChemPhysChem* **13**, 3714-3722 (2012).
5. Araneda J. F., Piers W. E., Heyne B., Parvez M., McDonald R., High stokes shift anilido-pyridine boron difluoride dyes. *Angew. Chem. Int. Ed.* **50**, 12214-12217 (2011).
6. Horváth P., Šebej P., Šolomek T., Klán P., Small-molecule fluorophores with large stokes shifts: 9-iminopyronin analogues as clickable tags. *J. Org. Chem.* **80**, 1299-1311 (2014).
7. Qian J., Ji W., Zhu H., Yang X., Yue H., Chen Q., He M., Zhang Z., Weak anionic ligands controlled synthesis of znii/cdii coordination polymers based on n-(4-pyridylmethyl)-l-threonine. *Polyhedron* **208**, 115411 (2021).
8. Zhang C., Yan Z. P., Dong X. Y., Han Z., Li S., Fu T., Zhu Y. Y., Zheng Y. X., Niu Y. Y., Zang S. Q., Enantiomeric mof crystals using helical channels as palettes with bright white circularly polarized luminescence. *Adv. Mater.* **32**, e2002914 (2020).
9. Tang X., Jiang H., Si Y., Rampal N., Gong W., Cheng C., Kang X., Fairen-Jimenez D., Cui Y., Liu Y., Endohedral functionalization of chiral metal-organic cages for encapsulating achiral dyes to induce circularly polarized luminescence. *Chem* **7**, 2771-2786 (2021).
10. Han X., Zhang J., Huang J., Wu X., Yuan D., Liu Y., Cui Y., Chiral induction in covalent organic frameworks. *Nat. Commun.* **9**, 1294 (2018).
11. Zhao T., Han J., Jin X., Liu Y., Liu M., Duan P., Enhanced circularly polarized

luminescence from reorganized chiral emitters on the skeleton of a zeolitic imidazolate framework. *Angew. Chem. Int. Ed.* **58**, 4978-4982 (2019).

12. Han Z., Wang K., Guo Y., Chen W., Zhang J., Zhang X., Siligardi G., Yang S., Zhou Z., Sun P., Shi W., Cheng P., Cation-induced chirality in a bifunctional metal-organic framework for quantitative enantioselective recognition. *Nat. Commun.* **10**, 5117 (2019).

13. Zhao T., Han J., Jin X., Zhou M., Liu Y., Duan P., Liu M., Dual-mode induction of tunable circularly polarized luminescence from chiral metal-organic frameworks. *Research* **2020**, 6452123 (2020).

14. Zeng M., Ren A., Wu W., Zhao Y., Zhan C., Yao J., Lanthanide mofs for inducing molecular chirality of achiral stilbazolium with strong circularly polarized luminescence and efficient energy transfer for color tuning. *Chem. Sci.* **11**, 9154-9161 (2020).

15. Wang X., Sun M., Huang Z., Xie M., Huang R., Lu H., Zhao Z., Zhou X., Li D., Turn-on circularly polarized luminescence in metal-organic frameworks. *Adv. Opt. Mater.* **9**, 2002096 (2021).

16. Wang Y., Yazawa K., Wang Q., Harada T., Shimoda S., Song Z., Bando M., Naga N., Nakano T., Optically active covalent organic frameworks and hyperbranched polymers with chirality induced by circularly polarized light. *Chem. Commun.* **57**, 7681-7684 (2021).

17. Zhang C., Li Z. S., Dong X. Y., Niu Y. Y., Zang S. Q., Multiple responsive cpl switches in an enantiomeric pair of perovskite confined in lanthanide mofs. *Adv. Mater.* **34**, e2109496 (2022).

18. Yu M. X., Liu C. P., Zhao Y. F., Li S. C., Yu Y. L., Lv J. Q., Chen L., Jiang F. L., Hong M. C., White-light emission and circularly polarized luminescence from a chiral copper(i) coordination polymer through symmetry-breaking crystallization. *Angew. Chem. Int. Ed.* **61**, e202201590 (2022).

19. Gao P. F., Jiang Y. Y., Liu H., Zhou M. S., Li T., Fu H. R., Ma L. F., Li D. S., Pillar-layer chiral mofs as a crystalline platform for circularly polarized luminescence and

single-phase white-light emission. *ACS Appl. Mater. Interfaces* **14**, 16435-16444 (2022).

20. Li Y., Ma X., Xu X., Ye Y., Wang B., Chiroptical activity of an achiral emissive eu metal-organic framework. *Chem. Eur. J.* **29**, e202203534 (2023).

21. Zhao T., Meng D., Hu Z., Sun W., Ji Y., Han J., Jin X., Wu X., Duan P., Enhanced chiroptic properties of nanocomposites of achiral plasmonic nanoparticles decorated with chiral dye-loaded micelles. *Nat. Commun.* **14**, 81 (2023).

22. Martens H., Naes T., *Multivariate calibration*. John Wiley & Sons: 1992.
